# Synthesis of *C*-glycosyl phosphonate derivatives of 4-amino-4-deoxy-α-ʟ-arabinose

**DOI:** 10.3762/bjoc.16.2

**Published:** 2020-01-02

**Authors:** Lukáš Kerner, Paul Kosma

**Affiliations:** 1 University of Natural Resources and Life Sciences, Vienna Department of Chemistry, Muthgasse 18, A-1190 Vienna, Austria

**Keywords:** antibiotic resistance, glycosyl phosphonate, glycosyl transferase, lipid A, lipopolysaccharide

## Abstract

The incorporation of basic substituents into the structurally conserved domains of cell wall lipopolysaccharides has been identified as a major mechanism contributing to antimicrobial resistance of Gram-negative pathogenic bacteria. Inhibition of the corresponding enzymatic steps, specifically the transfer of 4-amino-4-deoxy-ʟ-arabinose, would thus restore the activity of cationic antimicrobial peptides and several antimicrobial drugs. *C*-glycosidically-linked phospholipid derivatives of 4-amino-4-deoxy-ʟ-arabinose have been prepared as hydrolytically stable and chain-shortened analogues of the native undecaprenyl donor. The *C*-phosphonate unit was installed via a Wittig reaction of benzyl-protected 1,5-arabinonic acid lactone with the lithium salt of dimethyl methylphosphonate followed by an elimination step of the resulting hemiketal, leading to the corresponding *exo*- and *endo*-glycal derivatives. The ensuing selective monodemethylation and hydrogenolysis of the benzyl groups and reduction of the 4-azido group gave the α-ʟ-anomeric *arabino*- and *ribo*-configured methyl phosphonate esters. In addition, the monomethyl phosphonate glycal intermediates were converted into *n*-octyl derivatives followed by subsequent selective removal of the methyl phosphonate ester group and hydrogenation to give the octylphosphono derivatives. These intermediates will be of value for their future conversion into transition state analogues as well as for the introduction of various lipid extensions at the anomeric phosphonate moiety.

## Introduction

**Figure 1:**
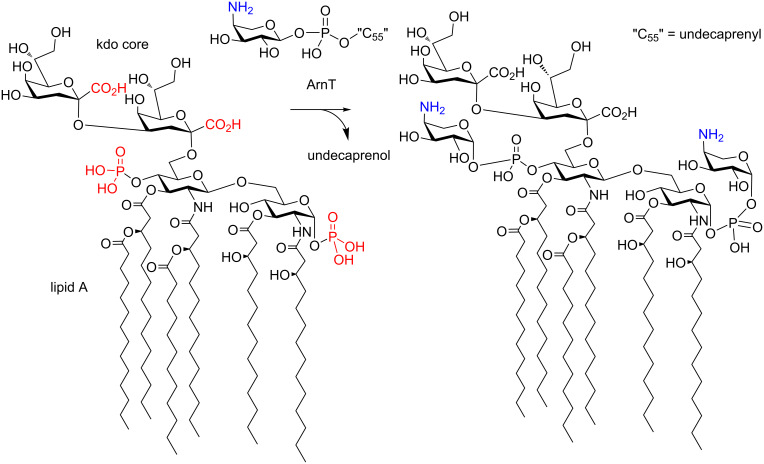
Modification of lipid A by ArnT.

Glycosyltransferases are important enzymes that accomplish the transfer of activated sugar phosphates onto their respective acceptor molecules [1]. In most cases, nucleotide diphosphate sugars serve as the reactive species, but lipid-linked diphosphate derivatives are equally important, e.g., when connected to dolichol in mammalian systems or to undecaprenol in prokaryotic donor substrates for bacterial glycosyltransferases [2]. 4-Amino-4-deoxy-ʟ-arabinose (Ara4N) is an important microbial carbohydrate in bacterial lipopolysaccharides (LPS) and has been implicated in resistance mechanisms of pathogenic Gram-negative bacteria against antibiotics, such as polymyxin B and colistin [3]. The main effect of Ara4N incorporation into the lipid A part – and, less frequently, into the inner core region of LPS [4] – is thought to originate from blocking the electrostatic interaction of cationic antimicrobial peptides with the negatively charged phosphate and carboxylate groups in LPS domains. Suitable inhibitors intercepting the attachment of Ara4N units to the lipid A and inner core region might restore sensitivity towards the cationic antimicrobial drugs as a novel approach to combat the looming antibiotic crisis [5]. 4-Amino-4-deoxy-ʟ-arabinose units are activated as the phosphodiester-linked undecaprenyl derivative [6], which is then transferred by the action of several Ara4N transferases (ArnT, Figure 1) [7].

The synthesis of potential inhibitors of the biosynthesis of Ara4N and the glycosyl transfer have not been fully explored yet. Previously, Kline and co-workers reported on the synthesis of acetylated 4-azido-arabinose phosphate and uridine diphosphate (UDP) derivatives. In addition, a 4-aminophosphoamidate UDP derivative was also obtained [8]. Whereas these compounds were inactive towards enzyme upstream of the biosynthetic pathway to undecaprenyl Ara4N, the peracetylated 4-azido derivative showed modest reduction of Ara4N incorporation into the lipid A part of *Salmonella typhimurium* [8].

We have recently set out to study the substrate specificity of ArnT enzymes in more detail using phosphodiester-linked derivatives in both anomeric configurations and containing short lipid appendages in order to define the minimum structural requirements for Ara4N glycosyltransferase substrates [9]. In parallel, we have started to develop *C*-phosphonate analogues as these have frequently been exploited as potential inhibitors for glycosyl transferases since the carbon–phosphorus bond is not hydrolyzed in the active site of glycosyl transferases [10–13]. Herein, we report on the synthesis of α-anomeric *C*-arabinosyl methylphosphonate ester derivatives as model compounds to allow for future incorporation of different lipid chains and options towards glycal analogues as potential transition state analogues to inhibit 4-amino-4-deoxy-ʟ-arabinose transfer to bacterial LPS.

## Results and Discussion

**Scheme 1:**
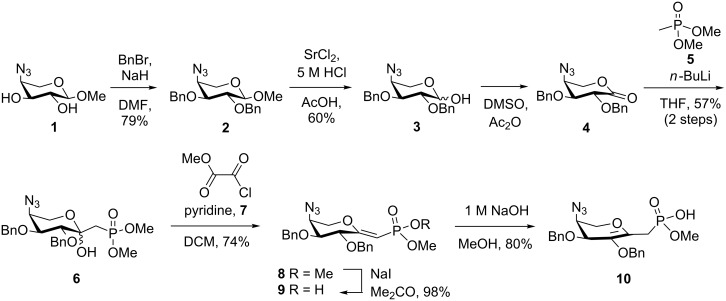
Phosphonate and glycal synthesis.

The previously synthesized [14] methyl 4-azido-4-deoxy-α-ʟ-arabinopyranoside (**1**), available in multigram amounts, was benzylated in 79% yield using benzyl bromide and sodium hydride in DMF, followed by hydrolysis of the anomeric aglycon of **2** with 5 M HCl and SrCl_2_ in acetic acid, which afforded the hemiacetal **3** as a 2:3 α/β anomeric mixture in 60% yield (Scheme 1). Conversion of benzylated reducing sugars, such as glucose [15], *N*-acetyl-ᴅ-glucosamine [16], and galactose [17], into the corresponding *C*-glycosyl phosponates using a Wittig reaction with methylenetriphenylphosphorane furnished the respective glycoenitols, which were then subjected to mercuriocyclization, followed by iodination and phosphonate introduction by an Arbusov reaction. Alternative approaches were elaborated from olefinic *C*-glycosides, which were converted into the corresponding *C*-linked hydroxymethyl derivatives and processed to give the glycosyl methylphosphonic acid derivatives [18–19]. As the Ara4N transferase reacted with the equatorial phosphodiester lipid, we opted to use lactone **4** as a suitable precursor for *C*-glycosyl phosphonates, based on literature precedents [10,20–21]. Thus, a modified Swern oxidation of lactol **3** with acetic anhydride in DMSO afforded a near-quantitative yield of lactone **4**, which, however, was unstable towards chromatographic purification and used as crude material after lyophilization to remove the solvent. Coupling of **4** with the lithium salt of dimethyl methylphosphonate (**5**) in THF afforded the β-anomeric ketol phosphonate derivative **6** in 57% yield. The axial orientation of the anomeric hydroxy group was proven by NOESY correlations between the H-2 proton and both geminal protons of the CH_2_P unit. A strong NOE was also observed for the upfield-shifted doublet of doublets at 1.71 ppm of the methylene group, which had an additional NOE correlation with the broad signal of the anomeric OH group at 5.79 ppm. Next, the ensuing elimination step was carried out to explore the access to transition state analogues [22] potentially mimicking the sp^2^ character of the oxocarbenium intermediate in the enzymatic transfer reaction. In addition, *exo*-glycals are versatile precursors for the introduction of fluoromethyl phosphonates, which are the better bioisosters of phosphates [23–25]. *Exo*-glycal **8** was obtained in 74% yield when using methyl oxalyl chloride [26], which proved to be superior to the use of trifluoroacetic anhydride, which gave **8** in 57% yield [27]. The *Z*-configuration of the hexenitol unit was derived from the ^3^*J*_C,P_ coupling constant (14.1 Hz), which was in good agreement with reported data [22,28]. Next, selective *O*-demethylation of the phosphonate diester was elaborated. Using sodium iodide in acetone afforded the mono-*O*-demethylated derivative **9** in 98% yield [29]. At this stage, the *Z*-configuration of the enol ether was unambiguously assigned on the basis of NOESY experiments. Specifically, NOE correlations were seen between the olefinic proton and the methylene protons of a benzyl group. The position of the 2-*O*-benzyl group was assigned on the basis of an HMBC correlation to position H-2 of the pyranose ring. Additional interactions were also found from the olefinic proton to hydrogen atom H-2 and the *O*-methyl group, respectively. The preferred formation of *Z*-configured Wittig products fully agreed with similar results in the literature for 2-*O*-benzyl-protected *gluco* and *galacto* derivatives [20,27,30–31].

In addition to exocyclic glycals mimicking putative planar transition states of substrates involved in enzymatic reactions, such as glycosyl transfer, mutase, and epimerization, *endo*-glycals are also of interest [21–22,32–35].

Under basic conditions, such as by treatment of **9** with 1 M NaOH in MeOH, the *exo* double bond of compound **9** could be shifted to produce the corresponding *endo*-glycal **10** in 80% yield. The *m*/*z* value of the high-resolution mass spectrum indicated that **10** was an isomer of **9**. In the ^1^H NMR (600 MHz) spectrum of **10**, the olefinic proton was absent, whereas the ^13^C NMR spectrum showed downfield shifts for the anomeric carbon atom (146.48 ppm) and the adjacent ring carbon atom (133.95 ppm). Evidence from an HMBC experiment additionally indicated a correlation between the benzylic protons and the latter carbon atom as well as signals of two upfield-shifted deoxy protons at 2.72 and 2.55 ppm, respectively, to the anomeric carbon atom. In addition, for compound **10**, lacking the conjugation to the phosphorus atom, a significant downfield shift of the ^31^P NMR signal was observed (17.97 ppm in **10** versus 10.95 ppm in compound **9**).

**Scheme 2:**
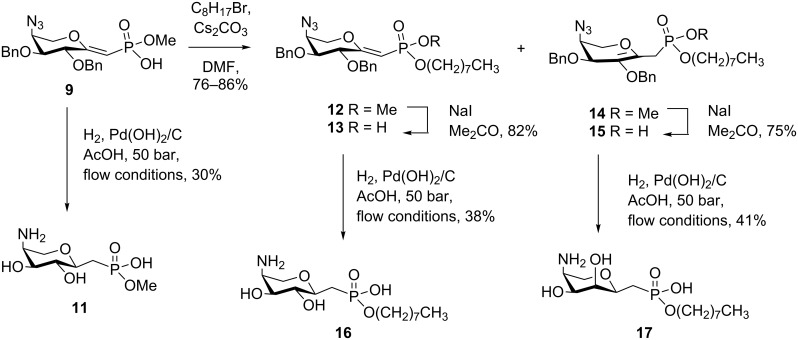
Synthesis of methyl phosphonate **11** and octyl phosphonates **16** and **17**.

Full deprotection, including the conversion of the 4-azido group into an amino function, was accomplished by hydrogenation of **9** in the presence of palladium hydroxide in a 1:1 mixture of methanol/acetic acid to deliver the target derivative **11** in 30% yield after final HILIC purification (Scheme 2). The equatorial arrangement of the *C*-glycosyl linkage was supported from the large value of the coupling constant *J*_1,2_ (9.5 Hz), indicating a 1,2-*trans* orientation of the respective protons.

The monomethyl phosphonate ester **9** was then subjected to alkylation reactions in order to allow for the selective introduction of longer-chain alkyl ester groups as better mimics of the native prenyl-activated donor substrate. Since the reaction of **9** with 1-bromooctane as model system involved basic conditions, mixtures of *endo*- and *exo*-glycal derivatives **12** and **14** were obtained. The highest yields were obtained for reactions performed in DMF, whereas DMSO and MeCN as solvents were less effective. Reaction times of 2–5 h at 80 °C were sufficient, the ratio of isomers obtained, however, critically depended on the work-up procedure. Addition of MeOH and silica gel in combination with dry loading of the column mainly produced the *endo*-glycal **14** as the major product (55%) as well as the *exo*-isomer (31%), since Cs_2_CO_3_ was still present to induce isomerization. In contrast, direct separation of a concentrated reaction mixture by HPLC afforded **12** as the major product (61%), with isolation of **14** as the minor isomer in 17% yield. The HPLC-based purification step even allowed for further separation of the diastereomers on phosphorus of both compounds.

Selective methyl ester cleavage of **12** and **14** – again using NaI in a minimum volume of acetone – was straightforward and furnished the octyl phosphonate ester derivatives **13** and **15** in good yields. For the future synthesis of deprotected glycal ester derivatives containing prenyl units, benzyl groups will have to be exchanged for protecting groups that can be removed under nonhydrogenolytic conditions, e.g., silyl ether protecting groups. In preliminary experiments, Lewis acid cleavage using BBr_3_, BCl_3_, and TiCl_4_, respectively, was not successful, although the latter reagent had previously been used to deprotect two benzyl groups from an allyl glycoside containing a 4-amino-4-deoxy-ʟ-arabinose residue [36]. Cleavage of the benzyl protecting groups with concomitant reduction of the azido group afforded the α-anomeric octyl ((4-amino-4-deoxy-ʟ-arabinopyranosyl)methyl)phosphonate **16** in 38% yield. Deprotection of the *endo*-glycal ester **15** was also investigated. An intermediate enol resulting from hydrogenolytic cleavage of the benzyl ether was envisaged to be prone to the facile formation of tautomers, which would then lead to different reduction products.

Notably, however, the reduction proved to be highly selective, and hence might have involved a fast hydrogen addition from the bottom face of the pyranose ring [37]. After full hydrogenation and azide-to-amine conversion, compound **17** was isolated as the 4-amino-4-deoxy-ʟ-ribopyranosyl derivative. The configuration was determined from the NMR data. Position H-2 at 4.02 ppm appeared as a broad doublet with small homonuclear coupling constants, as would be expected for a *manno* spin system. In addition, NOESY correlations were observed between atom H-3 and H-5ax as well as the anomeric proton, which was consistent with the equatorial position of the *C*-phosphonate entity.

## Conclusion

In conclusion, Wittig reactions of the suitably protected arabinonic lactone allowed for the straightforward implementation of a phosphonate dimethyl ester that could be readily converted into *exo*- and *endo*-glycal ester derivatives. The selective cleavage of one of the methyl groups opened various options for the introduction of a separate alkyl chain since the remaining methyl group was amenable to selective ester cleavage at a later stage. The glycal ester derivatives themselves offered additional options for the preparation of transition state analogues but can also be fully deprotected to provide hydrolytically stable substrate derivatives in the correct anomeric configuration.

## Supporting Information

File 1 Experimental section, spectral data, and copies of ^1^H, ^13^C, and ^31^P NMR spectra of compounds **4**, **6**, and **8**–**17**.
