# Supplementary material for: Synthesis of C-glycosyl phosphonate derivatives of 4-amino-4-deoxy-α-ʟ-arabinose
Source: Beilstein J Org Chem. 2020 Jan 2;16:9–14. doi: 10.3762/bjoc.16.2 (PMC6964659; doi:10.3762/bjoc.16.2)
Supplement: File 1 — Experimental section, spectral data, and copies of 1H, 13C, and 31P NMR spectra of compounds 4, 6, and 8–17. [file Beilstein_J_Org_Chem-16-09-s001.pdf]

BEILSTEIN JOURNAL OF ORGANIC CHEMISTRY

Supporting Information

for

Synthesis of *C*-glycosyl phosphonate derivatives of 4-amino-4-deoxy-α-L-arabinose

Lukáš Kerner and Paul Kosma

Beilstein J. Org. Chem. **2020,** *16,* 9–14. doi:10.3762/bjoc.16.2

Experimental section, spectral data, and copies of ^{1}H, ^{13}C, and ^{31}P NMR spectra of compounds 4, 6, and 8–17

License and Terms: This is a supporting information file under the terms of the Creative Commons Attribution License (https://creativecommons.org/licenses/by/4.0). Please note that the reuse, redistribution and reproduction in particular requires that the authors and source are credited.
The license is subject to the *Beilstein Journal of Organic Chemistry* terms and conditions: (https://www.beilstein-journals.org/bjoc)

Contents

General methods	S2
Experimental procedures and physico-chemical characterization	S2
References	S12
^{1}H, ^{13}C and ^{31}P NMR spectra	S13

General methods

Solvents and reagents were purchased from commercial suppliers and used as provided without further purification unless stated otherwise. Solvents (THF, CH_2Cl_2, DMF) were dried over activated 4 Å molecular sieves. Cation exchange resin DOWEX 50 H^+ was regenerated by consecutive washing with HCl (3 M), water and dry MeOH. If not indicated otherwise, concentration of organic solutions was performed under reduced pressure at <40 °C. Optical rotation was measured with an Anton Paar MCP100 Polarimeter at 20 °C. Reactions were followed by thin layer chromatography using Merck plates: generally on 5 × 10 cm, layer thickness 0.25 mm, Silica Gel $60F_{254}$; alternatively on HPTLC plates with 2.5 cm concentration zone (Merck). Spots were visualized with UV (254 nm) and/or anisaldehyde/H_2SO_4 staining. Preparative chromatography was performed using silica gel (0.040–0.063 mm) or a flash-purification system (Interchim, PuriFlash 4125). NMR spectra were recorded with a Bruker Avance III 600 instrument (^{1}H at 600 MHz, ^{13}C at 151 MHz and ^{31}P at 243 MHz) using standard Bruker NMR software. Chemical shifts are given in ppm down-field from $SiMe_4$ using the residual peak of $CDCl_3$ (7.26 for ^{1}H and 77.00 for ^{13}C), CD_3OD (3.31 for ^{1}H and 49.86 for ^{13}C) or D_2O (0.00 for ^{1}H, external calibration to 2,2-dimethyl-2-silapentane-5-sulfonic acid), 67.40 for ^{13}C (external calibration to 1,4-dioxane in D_2O), and orthophosphoric acid (δ = 0) for ^{31}P. Assignments for octyl signals are labeled with ‘. Numbering of phosphonate compounds was based on the nomenclature as hexit-1-yl derivatives. HRMS ESI-TOF data were obtained on a Waters Micromass Q-TOF Ultima Global instrument.

General conditions for hydrogenation of ***9**, **13*** *and* ***15:***

Hydrogenation was carried out in a ThalesNano H-Cube® reactor under the following conditions: flowrate 0.5 mL/min, cartridge length 30 mm, catalyst 20% $Pd(OH)_2/C$, temperature 50 °C, nominal H_2 pressure 50 bar. The starting material (typically 10 mg) was dissolved in 5–10 mL of a 1:1 MeOH/AcOH mixture and was run in loop for 2–3 h. After this time, the crude mixture was concentrated in vacuo, filtered over a syringe filter (0.45 µm, MeOH) and concentrated to dryness. All products were purified on a semi-preparative Merck SeQuant® ZIC®-HILIC (250 × 10 mm) HPLC column (MeCN → 1:1 MeCN/H_2O, 8 mL/min).

Methyl 4-azido-2,3-di-O-benzyl-4-deoxy-α-L-*arabino*pyranoside (2)

NaH (60% in mineral oil, 444 mg, 11.1 mmol, 2.1 equiv) was suspended in dry DMF (10 mL) and the mixture was cooled to 0 °C. A solution of **1**[1] (1 g, 5.29 mmol) in dry DMF (20 mL) was added dropwise under an inert atmosphere and stirring was continued at 0 °C for 30 min. Next, benzyl bromide (1.32 mL, 11.1 mmol, 2.1 equiv) was added dropwise, the reaction mixture was allowed to reach rt and stirring was continued for 22 h. The reaction was quenched by adding H_2O (20 mL) under cooling, which was followed by extraction with EtOAc (3 × 20 mL). Combined organic phases were washed with H_2O (20 mL), brine (20 mL), dried ($MgSO_4$) and concentrated in vacuo. The crude material was flash-chromatographed on silica gel (MePh → 1:1 MePh-EtOAc) to give 1.536 g (79%) of **2** as colorless oil, which solidified upon cooling. Physical and spectroscopic data were in agreement with literature.[2]

Oxidation of 4-azido-2,3-di-O-benzyl-4-deoxy-L-*arabino*pyranose (3)

Methyl glycoside **2** (100 mg, 0.27 mmol) was dissolved in a solution of $SrCl_2{\cdot}6H_2O$ (5 mg, 0.02 mmol, 0.07 equiv) in 1 mL of glacial AcOH and the mixture was warmed to70 °C. Diluted aq HCl (5 M, 0.16 mL, 0.81 mmol) was added dropwise and the resulting solution was stirred at 70 °C for 3 h and then for 70 h at rt. The reaction mixture was made neutral by adding a satd aq solution of $NaHCO_3$ (3 mL) and extracted with EtOAc (3 x 3 mL). Combined organic phases were dried ($MgSO_4$), concentrated *in vacuo* and flash-chromatographed on silica gel (4:1 MePh-EtOAc → EtOAc) to afford **3** as an oil which solidified upon standing. Yield: 58 mg (60%). NMR data matched published data.[2] The anomeric mixture was directly used without further separation. Lactol **3** (1.22 g, 3.43 mmol) was dissolved in DMSO (10 mL) followed by dropwise addition of acetic anhydride (8 mL, 85 mmol) under an inert atmosphere. The solution was stirred at rt overnight (22 h) and concentrated in vacuo at 50 °C. DMSO was removed by lyophilization and drying under high vacuum (<1 mbar) to give **4** (4-azido-2,3-di-*O*-benzyl-4-deoxy-L-*arabino*nic acid 1,5-lactone) as unstable brownish oil. Yield: 1.11 g (92%). R_f = 0.67 (4:1 MePh-EtOAc); $[\alpha]_D^{20}$ +87 (*c* 1.0, $CHCl_3$); ^1H NMR ($CDCl_3$): δ 7.34-7.29 (m, 10 H, H-Ar), 5.05 (d, 1 H, *J* = 11.2 Hz) and 4.68 (d, 1 H, *J* = 11.2 Hz, CH_2Ph), 4.74 (d, 1 H, *J* = 11.8 Hz) and 4.70 (d, 1 H, CH_2Ph), 4.33 4 (dd, 1 H, $J_{5a,5b}$ = 11.9 Hz, $J_{5a,4}$ = 5.2 Hz, H-5a), 4.25 (d, 1 H, $J_{2,3}$ = 7.4 Hz, H-2), 4.21 (dd, 1 H, $J_{5a,5b}$ = 11.9, $J_{5b,4}$ = 3.4 Hz Hz, H-5b), 4.04 (dt, 1 H, H-4),

3.99 (dd, 1 H, $J_{3,4}$ = 3.1, $J_{3,2}$ = 7.3 Hz, H-3); ^{13}C NMR ($CDCl_3$): δ 168.52 (C-1), 136.94 (d.i., C*q*, C-Ar), 128.59 (d.i.), 128.48 (d.i.), 128.40 (d.i.),128.20,128.16 and 127.85 (d.i., C-Ar), 77.58 (C-3), 76.16 (C-2),74.67 (*C*H$_2$Ph), 73.11 (*C*H$_2$Ph), 66.86 (C-5) and 56.47 (C-4). HRMS (ESI) *m/z* [M+H$^+$]$^+$ calcd for $C_{19}H_{19}N_3O_4$: 354.1448, found 354.1444.

Dimethyl (2,6-anhydro-5-azido-3,4-di-*O*-benzyl-1,5-dideoxy-β-L-*arabino*-hexulopyranosyl)phosphonate (6)

Dimethyl methyl phosphonate **5** (0.40 mL, 3.68 mmol, 1.3 equiv) was dissolved under an inert atmosphere in dry THF (5 mL) in a flame-pre-dried Schlenk flask and then cooled to −70 °C. Subsequently, 1.6 M solution of *n*-BuLi in hexanes (2.30 mL, 3.68 mmol, 1.3 equiv) was added dropwise into the flask and stirred for 30 min at −70 °C. A pre-cooled solution of lactone **4** (1.0 g, 2.83 mmol) in dry THF (5 mL) was added dropwise during 20 min, the resulting mixture was stirred at −70 °C for 1 h and then left to reach rt overnight (17 h). The reaction was quenched by adding MeOH (2 mL) and the solution was concentrated in vacuo. The crude residue was flash-chromatographed on silica gel (2:1 MePh/EtOAc → EtOAc) to give 773 mg (57%) of **6** as off-yellow syrup. R_f = 0.15 (2:1 MePh/EtOAc); $[\alpha]_D^{20}$ -14 (c 1.5, $CHCl_3$); ^{1}H NMR ($CDCl_3$): δ 7.41-7.29 (m, 10 H, H-Ar), 5.80 (br, s, 1 H, OH), 4.98 (d, 1 H, *J* = 11.6 Hz) and 4.66 (d, 1 H, *J* = 11.6 Hz, C*H$_2$*Ph), 4.76 (d, 2 H, C*H$_2$*Ph), 4.17 (dd, 1 H, $J_{4,5}$ = 3.7 Hz, $J_{3,4}$ = 9.5 Hz, H-4), 4.06 (dd, 1 H, $J_{6a,5}$ = 1.7 Hz, $J_{6a,6b}$ = 12.5 Hz, H-6a), 3.91 (ddd, 1 H, H-5), 3.71 (d, 3 H, $J_{Me,P}$ = 11.2 Hz, POC*H$_3$*), 3.64 (d, 3 H, $J_{Me,P}$ = 11.1 Hz, POC*H$_3$*), 3.59 (dd, 1 H, $J_{6b,5}$ = 1.7 Hz, $J_{6a,6b}$ = 12.5 Hz, H-6b), 3.56 (d, 1 H, $J_{4,3}$ = 9.5 Hz, H-3), 2.35 (dd, 1 H, $J_{CH2a,P}$ = 17.5 Hz, $J_{CH2a,CH2b}$ = 15.2 Hz, PCH$_2$a) and 1.71 (dd, 1 H, $J_{CH2b,P}$ = 18.6 Hz, $J_{CH2a,CH2b}$ = 15.2 Hz, PCH$_2$b); ^{13}C NMR ($CDCl_3$): δ 137.86 and 137.74 (C*q*, C-Ar), 128.74 (d.i.), 128.54 (d.i.), 128.46 (d.i.),128.00,127.96 and 127.93 (d.i., C-Ar), 97.41 (d, $J_{C,P}$ = 8.6 Hz, C-2), 78.92 (d, $J_{C,P}$ = 14.1 Hz, C-3), 78.49 (d, $J_{C,P}$ = 4.4 Hz, C-4), 75.36 (*C*H$_2$Ph), 72.76 (*C*H$_2$Ph), 61.21 (C-6), 60.44 (C-5), 53.30 (d, $J_{C,P}$ = 5.5 Hz, PO*C*H$_3$), 51.88 (d, $J_{C,P}$ = 6.5 Hz, PO*C*H$_3$), 32.31 (d, $J_{C,P}$ = 134.5 Hz, C-1); ^{31}P NMR ($CDCl_3$): δ 31.27. HRMS (ESI) *m/z* [M+H$^+$]$^+$ calcd for $C_{22}H_{28}N_3O_7P$: 478.1738, found 478.1735.

Dimethyl (2,6-anhydro-5-azido-3,4-di-*O*-benzyl-1,5-dideoxy-L-*arabino*-hex-1-enit-1-yl)phosphonate (8)

A solution of **6** (400 mg, 0.84 mmol) in dry DCM (15 mL) was cooled to 0 °C. Dry pyridine (3.38 mL, 41.88 mmol) was added dropwise under an inert atmosphere, followed by addition of methyl oxalyl chloride (0.77 mL, 8.38 mmol, 10 equiv), which resulted in immediate formation of a white precipitate. The suspension was stirred for 1 h at 0-5 °C, and then at rt for 23 h. The reaction mixture was diluted with DCM (15 mL) and treated with satd aq $NaHCO_3$ solution (20 mL) under cooling. The phases were separated and the aqueous portion was extracted with DCM (2 × 15 mL). The combined organic phases were dried ($MgSO_4$) and concentrated. The residue was purified by flash-chromatography on silica gel (1:1 MePh-EtOAc → EtOAc) to afford **8** as light brown oil. Yield: 286 mg (74%); R_f = 0.11 (1:1 MePh-EtOAc); $[\alpha]_D^{20}$ +54 (c 1.0, $CHCl_3$); ^1H NMR ($CDCl_3$): δ 7.35-7.29 (m, 10 H, H-Ar), 5.19 (dd, 1 H, $J_{=CH,2}$ = 0.8 Hz, $J_{=CH,P}$ = 12.6 Hz, =CH), 4.72 and 4.69 (2 d, each 1 H, *J* = 11.9 Hz, C*H₂*Ph), 4.69 and 4.61 (d, each 1 H, *J* = 11.5 Hz, C*H₂*Ph), 4.17 (dd, 1 H, $J_{6a,5}$ = 5.6 Hz, $J_{6a,6b}$ = 11.2 Hz, H-6a), 4.10 (br d, 1 H, $J_{4,3}$ = 7.1 Hz, H-3), 3.98 (dt, 1 H, $J_{4,5}$ = 3.2 Hz, H-5), 3.95 (dd, 1 H, $J_{6b,5}$ = 3.3 Hz, $J_{6a,6b}$ = 11.3 Hz, H-6b), 3.87 (dd, 1 H, $J_{4,3}$ = 7.1 Hz, $J_{5,4}$ = 3.2 Hz, H-4), 3.72 (d, 3 H, $J_{Me,P}$ = 3.5 Hz, POCH_3) and 3.70 (d, 3 H, $J_{Me,P}$ = 3.4 Hz, POCH_3); ^{13}C NMR ($CDCl_3$): δ 165.91 (d, $J_{C,P}$ = 1.6 Hz, =CH), 137.14 and 137.02 (C*q*, C-Ar), 128.55 (d.i.), 128.17, 128.07, 127.93 (d.i.) and 127.87 (d.i., Ar-C), 97.37 (d, $J_{C,P}$ = 189.5 Hz, C-2), 78.46 (C-4), 75.90 (d, $J_{C,P}$ = 14.1 Hz, C-3), 73.15 (*C*H_2Ph), 73.04 (*C*H_2Ph), 67.91 (C-6), 56.93 (C-5), 52.48 (d, $J_{C,P}$ = 5.5 Hz, PO*C*H_3), 52.15 (d, $J_{C,P}$ = 5.5 Hz, PO*C*H_3); ^{31}P NMR ($CDCl_3$): δ 19.05. HRMS (ESI) *m/z* $[M+H^+]^+$ calcd for $C_{22}H_{26}N_3O_6P$: 460.1632, found 460.1639.

Alternatively, **8** was prepared using TFAA:

Compound **6** (58 mg, 0.12 mmol) was dissolved in dry THF (1.5 mL) and cooled to 0°C. Dry pyridine (98 µL, 1.21 mmol) was added dropwise under an inert atmosphere, followed by trifluoroacetic anhydride (0.51 mL, 3.64 mmol, 30 equiv). After stirring at 0–5 °C for 90 min, the reaction mixture was allowed to reach rt and kept at this temperature for 27 h. Satd aq $NaHCO_3$ (5 mL) was added under cooling, which was followed by extraction with EtOAc (3 × 5 mL). Combined organic phases were dried ($MgSO_4$), concentrated and purified as described above to give 36 mg (57%) of **8** as turbid colorless oil.

Methyl (2,6-anhydro-5-azido-3,4-di-*O*-benzyl-1,5-dideoxy-L-*arabino*-hex-1-enit-1-yl)phosphonate (9)

Exo-glycal **8** (312 mg, 0.68 mmol) and NaI (204 mg, 1.36 mmol, 2 equiv) were mixed with dry acetone (5 mL). Under atmospheric pressure, the solution was concentrated at 70 °C to such an extent that stirring was still effective (≈1 h). The reaction vessel was then equipped with a septum and a balloon filled with Ar and the thick slurry was stirred at 60 °C overnight. The suspension was concentrated in vacuo and the residue was flash-chromatographed on silica gel (MePh/MeOH 2:1 → 1:2) to furnish **9** as yellow amorphous solid. Yield: 298 mg (98%), R_f = 0.39 (1:1 MePh-MeOH). $[\alpha]_D^{20}$ +32 (c 1.0, $CHCl_3$); ^{1}H NMR (CD_3OD): δ 7.37-7.26 (m, 10 H, H-Ar), 5.29 (d, 1 H, $J_{=CH,P}$ = 10.1 Hz, =CH), 4.74 and 4.62 (2 d, each 1 H, *J* = 11.5 Hz, C*H*$_2$Ph), 4.69 (d, 2 H, *J* = 11.7 Hz, C*H*$_2$Ph), 4.14-4.11 (m, 2 H, H-5, H-3), 4.09 (dd, 1 H, $J_{6a,5}$ = 5.6 Hz, $J_{6a,6b}$ = 11.5 Hz, H-6a), 3.91 (dd, 1 H, $J_{3,4}$ = 7.1 Hz, $J_{5,4}$ = 3.3 Hz H-4), 3.90 (dd, 1 H, $J_{6b,5}$ = 3.0 Hz, H-6b), 3.51 (d, 3 H, $J_{Me,P}$ = 11.2 Hz, POC*H*$_3$); ^{13}C NMR (CD_3OD): δ 163.01 (=CH), 140.11 and 140.08 (C*q*, C-Ar), 130.27 and 130.26 (3 C), 129.92, 129.86 (d.i.), 129.73, and 129.66 (Ar-C), 105.55 (d, $J_{C,P}$ = 176.3 Hz, C-2), 81.35 (C-4), 78.41 (d, $J_{C,P}$ = 12.9 Hz, C-3), 74.75 (*C*H$_2$Ph), 74.38 (*C*H$_2$Ph), 69.59 (C-6), 59.56 (C-5), 52.82 (d, $J_{C,P}$ = 5.1 Hz, PO*C*H$_3$); ^{31}P NMR (CD_3OD): δ 10.95. HRMS (ESI) *m/z* $[M+H^+]^+$ calcd for $C_{21}H_{24}N_3O_6P$: 446.1475, found 446.1482.

Methyl (2,6-anhydro-5-azido-3,4-di-*O*-benzyl-1,5-dideoxy-L-*erythro*-hex-2-enit-1-yl)phosphonate (10)

Exo-glycal **9** (50 mg, 0.11 mmol) was dissolved in MeOH (2 mL) and mixed with 1 M aq. NaOH solution (≈5.5 mL). The resulting suspension was stirred at 60–70 °C for 75 h. The reaction mixture was then concentrated in vacuo, filtered over Celite ($CHCl_3$) and the filtrate was concentrated. Yield: 40 mg (80%, crude) of **10** as brownish syrup. R_f = 0.28 (2:1 $CHCl_3$-MeOH). The crude material was repeatedly purified on silica gel ($CHCl_3$-MeOH = 2:1 → 1:1). $[\alpha]_D^{20}$ -48 (c 0.4, MeOH); ^{1}H NMR (CD_3OD): δ 7.38-7.24 (m, 10 H, H-Ar), 4.83 and 4.74 (2 d, each 1 H, *J* = 11.5 Hz, C*H*$_2$Ph), 4.73 (s, 2 H, C*H*$_2$Ph), 4.45 (app t, 1 H, $J_{5,4}$ = 3.8 Hz, H-4), 4.03 (ddd, 1 H, $J_{6a,4}$ = 1.0 Hz, $J_{6a,5}$ = 3.5 Hz, $J_{6a,6b}$ = 10.4 Hz, H-6a), 3.96 (t, 1 H, $J_{6b,5}$ = $J_{6a,6b}$ = 10.3 Hz, H-6b), 3.83 (dt, 1 H, H-5), 3.55 (d, 3 H, $J_{Me,P}$ = 10.7 Hz, POC*H*$_3$), 2.72 (dd, 1 H, *J* = 15.4 Hz, *J* = 20.2 Hz, H-1a), 2.55 (dd, 1 H, *J* = 14.8 Hz, *J* = 20.5 Hz, H-1b); ^{13}C NMR (CD_3OD): δ 146.48 (d, $J_{C,P}$ = 12.5 Hz, C-2), 140.55 and 140.00 (C*q*, C-Ar),

133.95 (d, $J_{C,P}$ = 10.6 Hz, C-3), 130.24 (d.i.), 130.22 (d.i.), 130.19 (d.i.), 130.08 (d.i.), 129.76 and 129.59 (Ar-C), 76.15 (*C*H_2Ph), 74.94 (*C*H_2Ph), 73.00 (C-4), 65.34 (C-6), 58.85 (C-5), 53.28 (d, $J_{C,P}$ = 5.9 Hz, PO*C*H_3), 29.11 (d, $J_{C,P}$ = 134.2 Hz, C-1); ^{31}P NMR (CD_3OD): δ 17.97. HRMS (ESI) *m/z* $[M+H^+]^+$ calcd for $C_{21}H_{24}N_3O_6P$: 446.1475, found 446.1474.

Methyl (2,6-anhydro-5-amino-1,5-dideoxy-L-*mannit*-1-yl) phosphonic acid (11)

Hydrogenation of **9** (30 mg, 0.067 mmol) was carried out as described under general conditions to give 4.9 mg (30%) of **11** as colorless oil after HILIC purification and drying in vacuo; $[\alpha]_D^{20}$ +32 (c 0.25, MeOH); ^{1}H NMR (D_2O): δ 3.87 (dd, 1 H, $J_{6a,5}$ = 1.1 Hz, $J_{6a,6b}$ = 13.6 Hz, H-6a), 3.77 (dd, 1 H, $J_{3,4}$ = 9.5 Hz, $J_{5,4}$ = 4.7 Hz, H-4), 3.62 (dd, 1 H, $J_{6b,5}$ = 1.2 Hz, $J_{6a,6b}$ = 13.6 Hz, H-6b), 3.51 (br d, 1 H, H-5), 3.395 (d, 3 H, $J_{Me,P}$ = 10.4 Hz, POC*H_3*), 3.325 (ddd, 1 H, $J_{2,3}$ = 9.7 Hz, $J_{2,1a}$ = 1.5 Hz, H-2) 3.25 (t, 1 H, $J_{2,3}$ = $J_{4,3}$ = 9.5 Hz, H-3), 2.02 (ddd, 1 H, $J_{1a,P}$ = 18.4 Hz, $J_{1a,1b}$ = 15.6 Hz, $J_{1a,12}$ = 2.5 Hz, H-1a), 1.68 (dt, 1 H, $J_{1b,P}$ = 15.5 Hz, $J_{1b,2}$ = 9.7 Hz, H-1b); ^{13}C NMR (D_2O): δ 77.92 (d, $J_{C,P}$ = 5.6 Hz, C-2), 71.96 (d, $J_{C,P}$ = 13.4 Hz, C-3), 70.69 (C-4), 66.62 (C-6), 53.19 (C-5), 52.04 (d, $J_{C,P}$ = 5.5 Hz, PO*C*H_3), and 29.17 (d, $J_{C,P}$ = 135.3 Hz, *C*H_2P); ^{31}P NMR (D_2O): δ 24.81. HRMS (ESI) *m/z* $[M+H^+]^+$ calcd for $C_7H_{16}NO_6P$: 242.0788, found 242.0786.

Methyl octyl (2,6-anhydro-5-azido-3,4-di-*O*-benzyl-1,5-dideoxy-L-*arabino*-hex-1-enit-1-yl)phosphonate (12) and Methyl octyl (2,6-anhydro-5-azido-3,4-di-*O*-benzyl-1,5-dideoxy-L-*erythro*-hex-2-enit-1-yl)phosphonate (14)

Method A

Phosphonate **9** (85 mg, 0.19 mmol) and Cs_2CO_3 (373 mg, 1.15 mmol) were suspended in dry DMF (2 mL) and warmed to 80 °C under an inert atmosphere. Then octyl bromide (99 µL, 0.57 mmol, 3 equiv) was added dropwise and the suspension was stirred for 2 h at 80 °C: The crude mixture was allowed to cool down and was concentrated under reduced pressure. The residue was suspended in MeOH, mixed with silica gel (≈2 g), concentrated in vacuo (40 °C) and the solid material was placed on top of a silica gel column and eluted with MePh → EtOAc, which gave 59 mg (55%) of **14** and 33 mg (31%) of **12** as yellow oils.

Method B

Phosphonate **9** (100 mg, 0.225 mmol) and Cs_2CO_3 (439 mg, 1.374 mmol, 6 equiv) were suspended in dry DMF (2.8 mL) and reacted with octyl bromide (116 µL, 0.674 mmol) as described above. The mixture was brought to rt, concentrated under reduced pressure and directly flash-chromatographed on silica gel (MePh → EtOAc) to afford 21 mg (17%) of **14** and 76 mg (61%) of **12** as off-yellow syrups. Resolution of diastereomers of **12** and **14** was achieved on a semi-preparative YMC Pack Sil 06 (250 × 20 mm) HPLC column (MePh → EtOAc, 25 mL/min) to give **14a** as transparent oil, R_f = 0.60 (EtOAc); $[\alpha]_D^{20}$ -50 (*c* 0.95, $CHCl_3$) followed by **14b** as tranparent oil, R_f = 0.56 (EtOAc); $[\alpha]_D^{20}$ -450 (*c* 1.16, $CHCl_3$,); ^{1}H NMR ($CDCl_3$): δ 7.38-7.27 (m, 10 H, H-Ar), 4.845 and 4.75 (2 d, each 1 H, *J* = 11.5 Hz, CH_2Ph), 4.72 and 4.68 (2 d, each 1 H, *J* = 11.1 Hz, CH_2Ph), 4.28 (app t, 1 H, $J_{5,4}$ = 3.9 Hz, H-4), 4.05 (d, 2 H, H-6a, H-6b), 4.01 (q, 2 H, *J* = 7.0 Hz, H-1'a, H-1'b of **14a**), 4.00 (q, 2 H, *J* = 7.0 Hz, H-1'a, H-1'b of **14b**), 3.73 (d, 3 H, $J_{Me,P}$ = 11.0 Hz, $POCH_3$ of **14b**), 3.70 (d, 3 H, $J_{Me,P}$ = 11.0 Hz, $POCH_3$ of **14a**), 3.69 (ddd, 1 H, $J_{5,4}$ = 3.9 Hz, H-5), 2.915 (dd, 1 H, *J* = 15.1 Hz, *J* = 21.3 Hz, H-1a), 2.71 (dd, 1 H, *J* = 15.1 Hz, *J* = 21.2 Hz, H-1b), 1.66-160 (m, 2 H, *J* = 6.7 Hz, H-2'a, H-2'b), 1.34-1.26 (m, 10 H, octyl signals H-3', H-4', H-5', H-6', H-7') and 0.88 (t, 3 H, *J* = 6.9 Hz, H-8'); ^{13}C NMR ($CDCl_3$) for **14a**: δ 141.16 (d, $J_{C,P}$ = 12.9 Hz, C-2), 137.86 and 137.23 (C*q*, C-Ar), 133.26 (d, $J_{C,P}$ = 11.7 Hz, C-3), 128.47 (d.i.), 128.41 (d.i.), 128.03 (d.i.), 128.01, 127.92 (d.i.) and 127.83 (Ar-C), 74.51 (d, $J_{C,P}$ = 3.1 Hz, 2-O*C*H_2Ph), 73.21 (*C*H_2Ph), 70.48 (C-4) 66.36 (d, $J_{C,P}$ = 6.5 Hz, C-1'), 63.23 (C-6), 56.82 (C-5), 52.52 (d, $J_{C,P}$ = 6.5 Hz, $POCH_3$), 31.76 (C-6'), 30.51 (d, $J_{C,P}$ = 6.4 Hz, C-2'), 29.18 and 29.12 (C-4', C-5'), 26.22 (d, $J_{C,P}$ = 139.6 Hz, C-1), 25.46 (C-3'), 22.61 (C-7') and 14.05 (C-8'); ^{13}C NMR ($CDCl_3$) for **14b**: δ 141.13 (d, $J_{C,P}$ = 13.0 Hz, C-2), 137.86 and 137.22 (C*q*, C-Ar), 133.30 (d, $J_{C,P}$ = 11.9 Hz, C-3), 128.46 (d.i.), 128.41 (d.i.), 128.03 (d.i.), 128.02, 127.93 (d.i.) and 127.83 (Ar-C), 74.49 (d, $J_{C,P}$ = 3.1 Hz, 2-O*C*H_2Ph), 73.20 (*C*H_2Ph), 70.41 (C-4), 66.21 (d, $J_{C,P}$ = 6.6 Hz, C-1'), 63.25 (C-6), 56.80 (C-5), 52.70 (d, $J_{C,P}$ = 6.5 Hz, $POCH_3$), 31.76 (C-6'), 30.48 (d, $J_{C,P}$ = 5.8 Hz, C-2'), 29.16 and 29.12 (C-4', C-5'), 26.26 (d, $J_{C,P}$ = 139.6 Hz, C-1), 25.45 (C-3'), 22.61 (C-7') and 14.06 (C-8') ^{31}P NMR ($CDCl_3$): δ 25.65 (**14b**) and 25.62 (**14a**). HRMS (ESI) *m/z* $[M+H^+]^+$ calcd for $C_{29}H_{40}N_3O_6P$: 558.2727, found 558.2734 for **14a**; found 558.2740 for **14b**.

Continued elution of the column afforded **12a** as transparent oil, R_f = 0.34 (EtOAc); $[\alpha]_D^{20}$ +40 (*c* 1.12, $CHCl_3$,) and **12b** R_f = 0.34 (EtOAc); $[\alpha]_D^{20}$ +42 (*c* 1.20, $CHCl_3$,);

^{1}H NMR ($CDCl_3$): δ 7.36-7.29 (m, 10 H, H-Ar), 5.20 (d, 1 H, $J_{=CH,P}$ = 12.5 Hz, =CH of **12b**), 5.19 (d, 1 H, $J_{=CH,P}$ = 12.4 Hz, =CH of **12b**), 4.70 (br s, 2 H, C*H2*Ph), 4.69 and 4.59 (2 d, each 1 H, *J* = 11.5 Hz, C*H2*Ph), 4.15 (dd, 1 H, $J_{6a,5}$ = 5.7 Hz, $J_{6a,6b}$ = 11.1 Hz, H-6a), 4.09 (br d, 1 H, $J_{3,4}$ = 7.0 Hz, H-3), 4.01-3.97 (m, 3 H, H-4, H-1'a, H-1'b), 3.95 (dd, 1 H, $J_{6b,5}$ = 3.4 Hz, $J_{6a,6b}$ = 11.1 Hz, H-6b of **12a**), 3.93 (dd, 1 H, $J_{6b,5}$ = 3.5 Hz, $J_{6a,6b}$ = 11.4 Hz, H-6b of **12b**), 3.87 (dd, 1 H, $J_{4,3}$ = 7.0 Hz, $J_{5,4}$ = 3.1 Hz, H-4 of **12a**), 3.86 (dd, 1 H, $J_{4,3}$ = 7.1 Hz, $J_{5,4}$ = 3.3 Hz, H-4 of **12b**), 3.71 (d, 3 H, $J_{Me,P}$ = 11.4 Hz, POC*H3* of **12a**), 3.70 (d, 3 H, $J_{Me,P}$ = 11.3 Hz, POC*H3* of **12b**), 1.64 (dt, 2 H, *J* = 6.8 Hz, H-2'a, H-2'b), 1.36-1.24 (m, 10 H, octyl signals H-3‘, H-4‘, H-5‘, H-6‘, H-7‘) and 0.88 (t, 3 H, *J* = 7.1 Hz, H-8‘); ^{13}C NMR ($CDCl_3$) for **12a**: δ 165.40 (=CH), 137.17 and 137.07 (C*q*, C-Ar), 128.56 (d.i.), 128.17, 128.07, 127.93 (d.i.) and 127.87 (d.i., Ar-C), 98.78 (d, $J_{C,P}$ = 188.7 Hz, C-2), 78.41 (C-4), 75.95 (d, $J_{C,P}$ = 14.7 Hz, C-3), 73.04 (*C*H2Ph), 73.03 (*C*H2Ph), 67.78 (C-6), 65.72 (d, $J_{C,P}$ = 5.7 Hz, C-1‘) 56.86 (C-5), 52.39 (d, $J_{C,P}$ = 5.8 Hz, PO*C*H3), 31.77 (C-6‘), 30.50 (d, $J_{C,P}$ = 6.3 Hz, C-3‘), 29.18 and 29.15 (C-4‘, C-5‘), 25.55 (C-3‘), 22.62 (C-7‘) and 14.06 (C-8‘); ^{13}C NMR ($CDCl_3$) for **12b**: δ 165.47 (=CH), 137.18 and 137.07 (C*q*, C-Ar), 128.56, 128.55, 128.16, 128.07, 127.93 (d.i.) and 127.88 (d.i., Ar-C), 98.16 (d, $J_{C,P}$ = 188.9 Hz, C-2), 78.49 (C-4), 75.89 (d, $J_{C,P}$ = 14.1 Hz, C-3), 73.11 (*C*H2Ph), 73.04 (*C*H2Ph), 67.83 (C-6), 65.99 (d, $J_{C,P}$ = 6.1 Hz, C-1‘) 56.96 (C-5), 52.07 (d, $J_{C,P}$ = 5.5 Hz, PO*C*H3), 31.77 (C-6‘), 30.51 (d, $J_{C,P}$ = 6.6 Hz, C-3‘), 29.18 and 29.15 (C-4‘, C-5‘), 25.55 (C-3‘), 22.62 (C-7‘) and 14.06 (C-8‘); ^{31}P NMR ($CDCl_3$): δ 17.53 (**12b**) and 17.52 (**12a**). HRMS (ESI) *m/z* $[M+H^+]^+$ calcd for $C_{29}H_{40}N_3O_6P$: 558.2727, found 558.2739 for **12a**; found 558.2740 for **12b**.

Octyl (2,6-anhydro-5-azido-3,4-di-*O*-benzyl-1,5-dideoxy-L-*arabino*-hex-1-enit-1-yl)phosphonate (13)

Dry acetone (2 mL) was added to *exo*-glycal **12** (75 mg, 0.13 mmol) and NaI (81 mg, 0.54 mmol, 4 equiv). The suspension was concentrated at 70 °C at atmospheric pressure to such an extent that stirring was still effective (≈30 min). The reaction vessel was then equipped with a septum and a balloon filled with Ar and the thick slurry was stirred at 55 °C overnight (19 h). Then another portion of NaI was added (40 mg, 0.27 mmol, 2 equiv) and stirring was continued for additional 47 h. The suspension was concentrated in vacuo and subjected to flash-chromatography on silica gel ($CHCl_3$/MeOH 9:1 → 1:1) to give 60 mg (82%) of **13** as yellow oil. R_f = 0.17

(EtOAc); $[\alpha]_D^{20}$ +31 (*c* 1.2, MeOH); ^{1}H NMR (CD_3OD): δ 7.36-7.26 (m, 10 H, H-Ar), 5.29 (d, 1 H, $J_{=CH,P}$ = 10.0 Hz, =CH), 4.73 and 4.69 (2 d, each 1 H, *J* = 11.6 Hz, C*H₂*Ph), 4.68 and 4.61 (2 d, each 1 H, *J* = 11.5 Hz, C*H₂*Ph), 4.13-4.11 (m, 2 H, H-5, H-3), 4.08 (dd, 1 H, $J_{6a,5}$ = 5.9 Hz, $J_{6a,6b}$ = 11.2 Hz, H-6a), 3.93 (dd, 1 H, $J_{3,4}$ = 7.1 Hz, $J_{5,4}$ = 3.2 Hz, H-4), 3.91 (dd, 1 H, $J_{6b,5}$ = 3.1 Hz, H-6b), 3.77 (dt, *J* = 6.5 Hz, H-1'a, H-1'b), 1.55 (m, 2 H, *J* = 6.7 Hz, H-2'a, H-2'b), 1.35-1.24 (m, 10 H, octyl signals H-3', H-4', H-5', H-6', H-7') and 0.88 (t, 3 H, *J* = 6.9 Hz, H-8'); ^{13}C NMR (CD_3OD): δ 162.70 (by HMBC, C-2), 140.11 and 140.06 (C*q*, C-Ar), 130.28 and 130.26 (3 C), 129.94 (d.i.), 129.83 (d.i.), 129.75, and 129.67 (Ar-C), 105.9 (by HMBC, C-1), 81.18 (C-4), 78.40 (d, $J_{C,P}$ = 12.8 Hz, C-3), 74.60 (*C*H_2Ph), 74.39 (*C*H_2Ph), 69.42 (C-6), 66.41 (C-1'), 59.30 (C-5), 33.87 (C-6'), 32.91 (C-2'), 31.35 and 31.31 (C-4', C-5'), 27.89 (C-3'), 24.58 (C-7') and 15.28 (C-8'); ^{31}P NMR (CD_3OD): δ 9.06. HRMS (ESI) *m/z* $[M+H^+]^+$ calcd for $C_{28}H_{38}N_3O_6P$: 544.2571, found 544.2577.

Octyl (2,6-anhydro-5-azido-3,4-di-*O*-benzyl-1,5-dideoxy-L-*erythro*-hex-2-enit-1-yl)phosphonate (15)

Dry acetone (1 mL) was added to *endo*-glycal **14** (22 mg, 0.039 mmol) and NaI (24 mg, 0.158 mmol, 4 equiv). The suspension was concentrated at atmospheric pressure so that stirring was still effective and the resulting slurry was stirred at 60 °C overnight (16 h) under Ar. Then another portion of NaI was added (24 mg, 0.158 mmol, 4 equiv) and stirring was continued for additional 28 h. The reaction mixture was concentrated in vacuo and flash-chromatographed on silica gel ($CHCl_3$/MeOH = 9:1 → 1:1) to afford **15** as off-yellow syrup. Yield: 16 mg (75%); R_f = 0.25 (EtOAc); $[\alpha]_D^{20}$ -50 (c 0.44, $CHCl_3$); ^{1}H NMR ($CDCl_3$): δ 7.38-7.24 (m, 10 H, H-Ar), 4.85 and 4.75 (2 d, each 1 H, *J* = 11.5 Hz, C*H₂*Ph), 4.73 and 4.70 (2 d, each 1 H, *J* = 11.1 Hz, C*H₂*Ph), 4.28 (br t, 1 H, H-4), 4.02 (dd, 1 H, $J_{6a,5}$ = 3.2 Hz, $J_{6a,6b}$ = 10.4 Hz, H-6a), 3.96 (t, 1 H, $J_{6b,5}$ = $J_{6a,6b}$ = 10.2 Hz, H-6b), 3.85-3.81 (m, 3 H, H-5, H-1'a, H-1'b), 1.68-1.58 (m, 2 H, *J* = 6.7 Hz, H-2'a, H-2'b), 1.33-1.22 (m, 10 H, octyl signals H-3', H-4', H-5', H-6', H-7') and 0.88 (t, 3 H, *J* = 7.2 Hz, H-8'); ^{13}C NMR ($CDCl_3$): δ 146.53 (d, $J_{C,P}$ = 11.0 Hz, C-2), 140.56 and 139.94 (C*q*, C-Ar), 133.96 (d, $J_{C,P}$ = 9.6 Hz, C-3), 130.25 (d.i.), 130.20 (d.i.), 130.19 (d.i.), 130.03 (d.i.), 129.80 and 129.59 (Ar-C), 76.13 (*C*H_2Ph), 74.96 (*C*H_2Ph), 73.01 (C-4), 66.84 (C-1'), 65.36 (C-6), 58.90 (C-5), 33.87 (C-6'), 32.97 (d, $J_{C,P}$ = 6.6 Hz, C-2'), 31.38 and 31.32 (C-4', C-5'), 29.6 (C-1 by

HSQC), 27.84 (C-3‘), 24.58 (C-7‘), 15.29 (C-8‘); ^{31}P NMR ($CDCl_3$): δ 16.19. HRMS (ESI) *m/z* [M+H$^+$]$^+$ calcd for $C_{28}H_{38}N_3O_6P$: 544.2571, found 544.2579.

Octyl (2,6-anhydro-5-amino-1,5-dideoxy-L-*mannit-1-yl*) phosphonic acid (16)
Hydrogenation of **13** (13 mg, 0.024 mmol) was carried out as described under general conditions to give 3.1 mg (38%) of **16** as colorless oil after HILIC purification; $[\alpha]_D^{20}$ +27 (c 0.3, MeOH); ^{1}H NMR (CD_3OD): δ 3.88 (dd, 1 H, $J_{6a,5}$ = 1.6 Hz, $J_{6a,56b}$ = 13.0 Hz, H-6a), 3.85 (q, 2 H, *J* = 6.5 Hz, POC*H*$_2$), 3.77-3.64 (m, 2 H, H-6b, H-4), 3.45-3.39 (m, 2 H, H-2, H-3), 3.28 (br, d, 1 H, $J_{5,4}$ = 4.4 Hz, H-5), 2.09 (ddd, 1 H, $J_{1a,P}$ = 17.6 Hz, $J_{1a,1b}$ = 15.3 Hz, $J_{1a,2}$ = 4.8 Hz, H-1a), 1.91 (ddd, 1 H, $J_{1b,P}$ = 17.3 Hz, $J_{1b,2}$ = 6.0 Hz, $J_{1a,1b}$ = 15.2 Hz, H-1b), 1.64-1.59 (m, 2 H, *J* = 6.7 Hz, H-2’a, H-2’b), 1.41-1.28 (m, 10 H, octyl signals H-3‘, H-4‘, H-5‘, H-6‘, H-7‘) and 0.90 (t, 3 H, *J* = 7.0 Hz, H-8‘); ^{13}C NMR (CD_3OD): δ 79.74 (d, $J_{C,P}$ = 2.8 Hz, C-2), 74.30 (d, $J_{C,P}$ = 7.8 Hz, C-3), 73.86 (C-4), 69.09 (C-6), 66.13 (d, $J_{C,P}$ = 5.6 Hz, C-1‘), 54.49 (C-5), 33.87 (C-6‘), 32.76 (d, $J_{C,P}$ = 134.0 Hz, C-1), 33.04 and 33.00 (C-4‘, C-5‘), 27.86 (C-3‘), 24.57 (C-7‘) and 15.26 (C-8‘); ^{31}P NMR (CD_3OD): δ 21.56. HRMS (ESI) *m/z* [M+H$^+$]$^+$ calcd for $C_{14}H_{30}NO_6P$: 340.1884, found 340.1887.

Octyl (2,6-anhydro-5-amino-1,5-dideoxy-L-*altrit*-1-yl) phosphonic acid (17)
Hydrogenation of **15** (9 mg, 0.017 mmol) was carried out as described under general conditions to give 2.3 mg (41%) of **17** as white solid after HILIC purification; $[\alpha]_D^{20}$ +43 (*c* 0.2, MeOH); ^{1}H NMR (CD_3OD): δ 4.02 (br d, 1 H, H-3), 4.01 (br d, 1 H, $J_{6a,5}$ = 1.4 Hz, $J_{6a,6b}$ = 13.4 Hz, H-6a), 3.84 (m, 3 H, *J* = 8.6 Hz, POC*H*$_2$, H-4), 3.71 (br d, 1 H, $J_{6a,6b}$ = 13.5 Hz, H-6b), 3.68 (ddd, 1 H, $J_{2,P}$ = 5.6 Hz, $J_{2,1a}$ = 8.3 Hz, $J_{2,1b}$ = 5.3 Hz, H-2), 3.32 (br d, 1 H, *J* = 2.5 Hz, H-5), 2.05 (ddd, 1 H, $J_{1a,P}$ = 16.9 Hz, $J_{1a,1b}$ = 14.9 Hz, $J_{1a,2}$ = 8.2 Hz, H-1a), 1.93 (ddd, 1 H, $J_{1b,P}$ = 18.4 Hz, $J_{1b,2}$ = 5.3 Hz, H-1b), 1.64-1.59 (m, 2 H, *J* = 6.7 Hz, H-2’a, H-2’b), 1.40-1.30 (m, 10 H, octyl signals H-3‘, H-4‘, H-5‘, H-6‘, H-7‘) and 0.90 (t, 3 H, *J* = 7.1 Hz, H-8‘); ^{13}C NMR (CD_3OD): δ 78.44 (C-2), 72.44 (d, $J_{C,P}$ = 4.95 Hz, C-3), 68.67 (C-4), 68.62 (C-6), 66.03 (d, $J_{C,P}$ = 6.1 Hz, C-1‘), 53.76 (C-5), 33.86 (C-6‘), 33.01 (d, $J_{C,P}$ = 6.6 Hz, C-2‘), 31.40 (d, $J_{C,P}$ = 133.1 Hz, *C*H_2P), 31.34 and 31.29 (C-4‘, C-5‘), 27.85 (C-3‘), 24.56 (C-7‘), 15.26 (C-8‘); ^{31}P NMR (CD_3OD): δ 20.56. HRMS (ESI) *m/z* [M+H$^+$]$^+$ calcd for $C_{14}H_{30}NO_6P$: 340.1884, found 340.1887.

References

1. B. Müller, M. Blaukopf, A. Hofinger, A. Zamyatina, H. Brade, P. Kosma, *Synthesis* **2010**, 3134-3151.

2. M. Blaukopf, B. Müller, A. Hofinger, P. Kosma, *Eur. J. Org. Chem*. **2012**, 119-131.

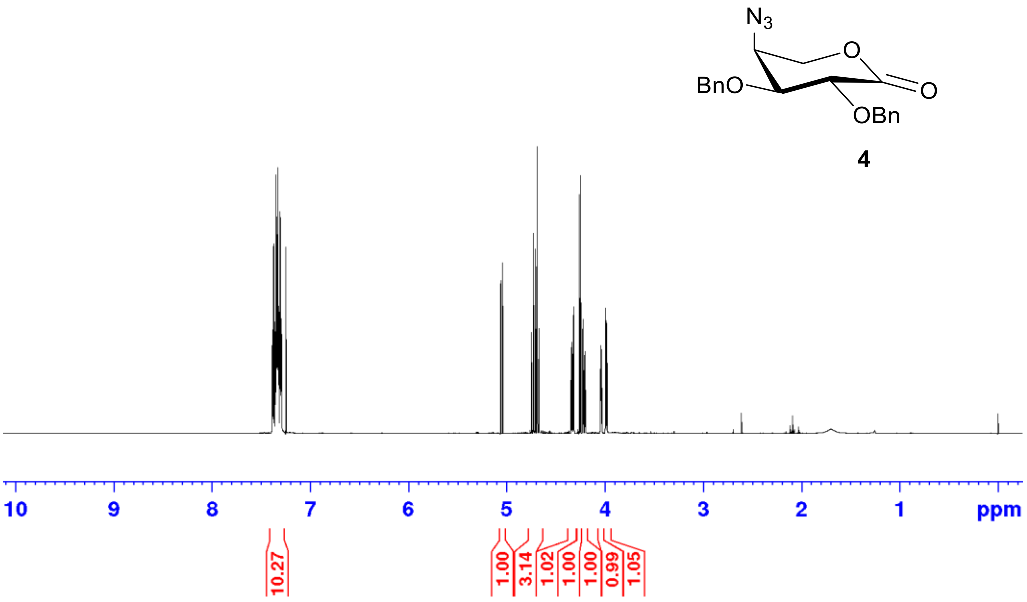
N3
O
BnO
O
OBn
4
10
9
8
7
6
5
4
3
2
1
ppm
10.27
1.00
3.14
1.02
1.00
1.00
0.99
1.05

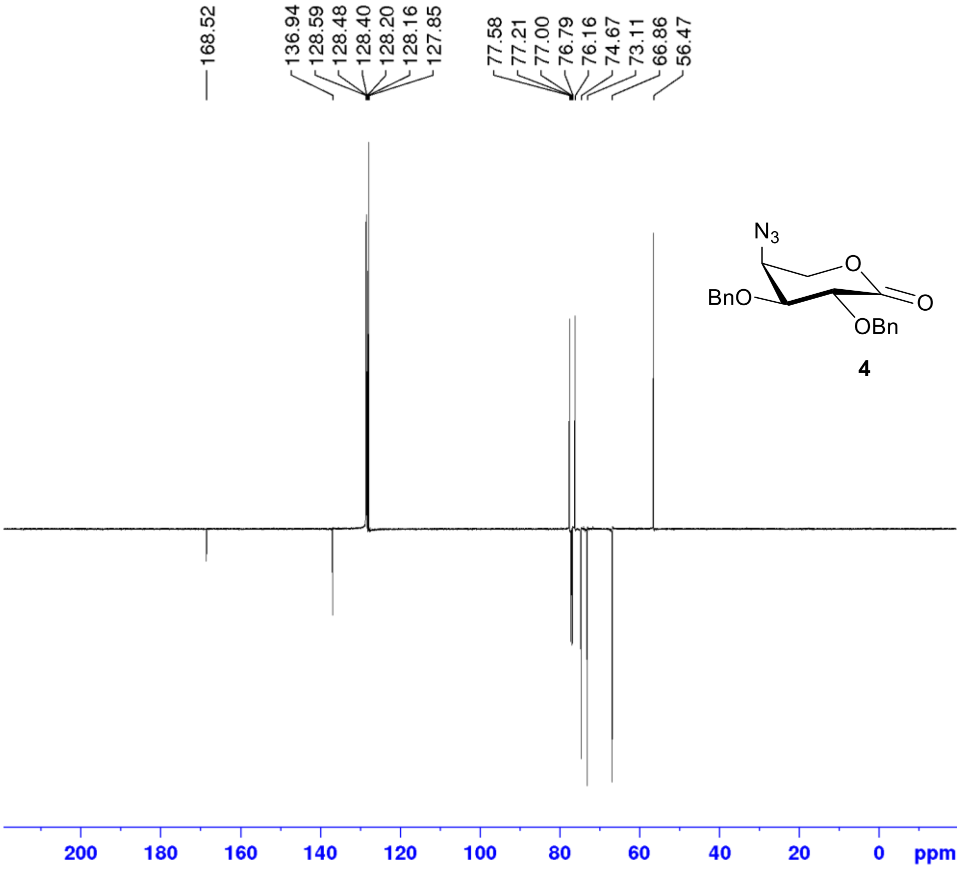
168.52
136.94
128.59
128.48
128.40
128.20
128.16
127.85
77.58
77.21
77.00
76.79
76.16
74.67
73.11
66.86
56.47
N3
O
BnO
O
OBn
4
200
180
160
140
120
100
80
60
40
20
0
ppm

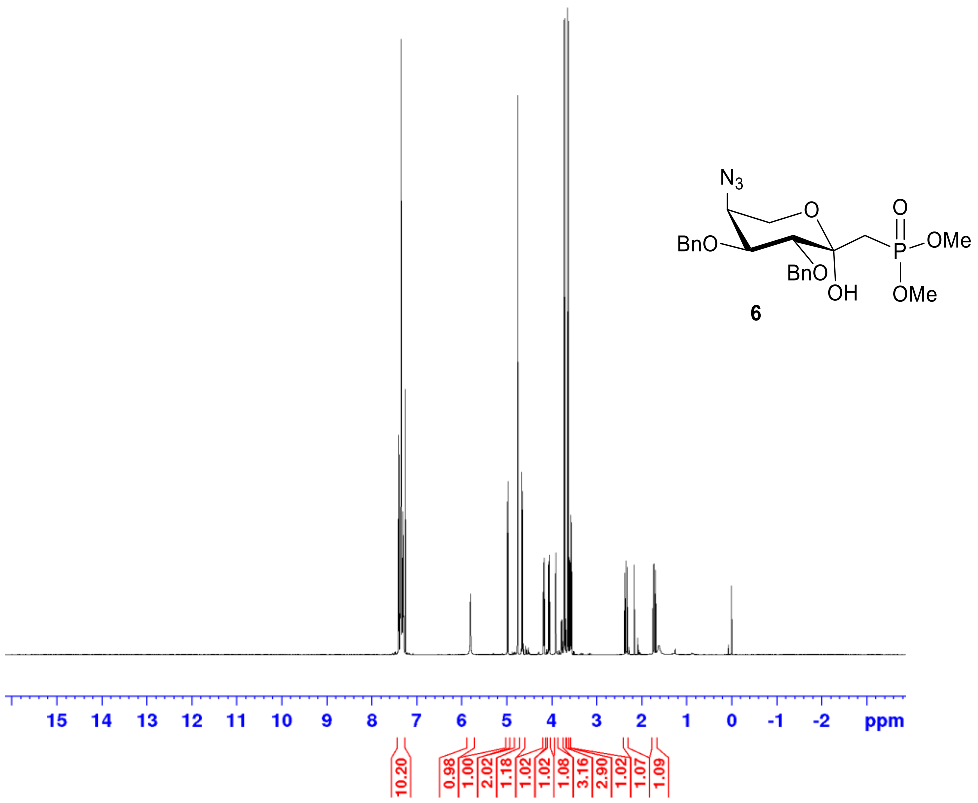
N3
BnO
O
O
P
OMe
BnO
OH
OMe
6
15 14 13 12 11 10 9 8 7 6 5 4 3 2 1 0 -1 -2 ppm
10.20
0.98 1.00 2.02 1.18 1.02 1.02 1.08 3.16 2.90 1.02 1.07 1.09

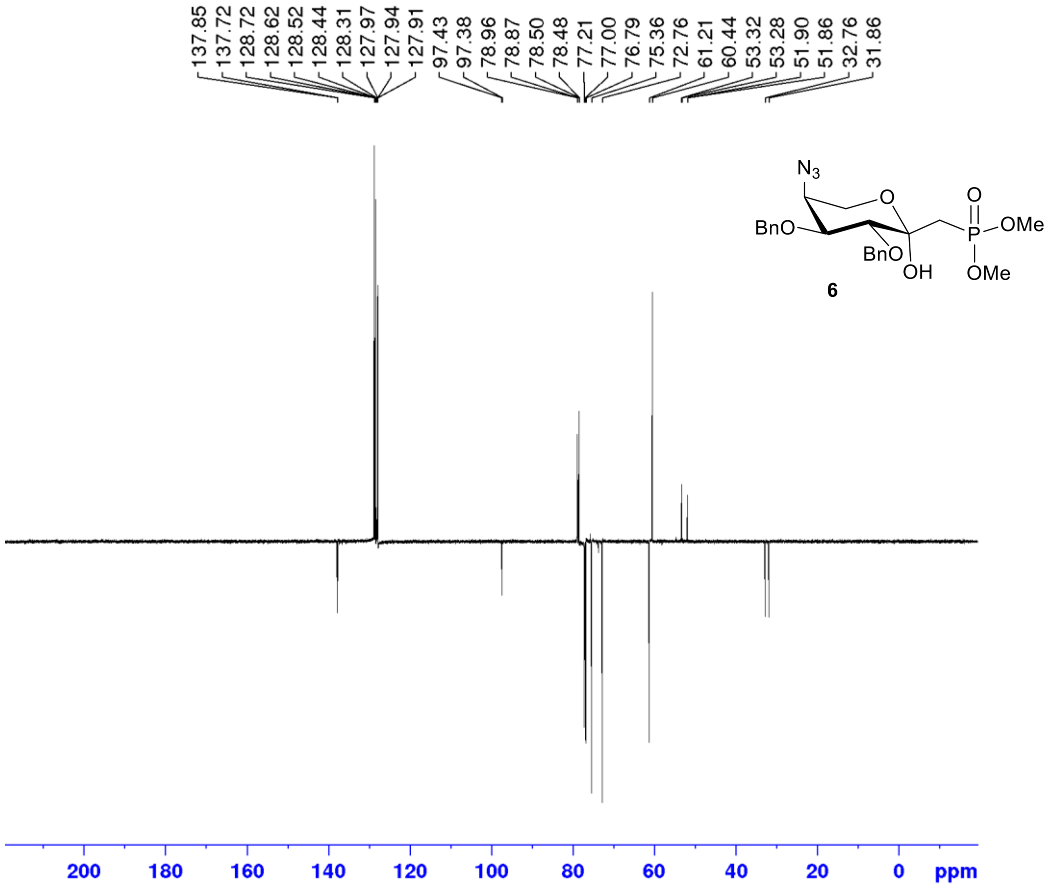
137.85 137.72 128.72 128.62 128.52 128.44 128.31 127.97 127.94 127.91 97.43 97.38 78.96 78.87 78.50 78.48 77.21 77.00 76.79 75.36 72.76 61.21 60.44 53.32 53.28 51.90 51.86 32.76 31.86
N3
BnO
O
O
P
OMe
BnO
OH
OMe
6
200 180 160 140 120 100 80 60 40 20 0 ppm

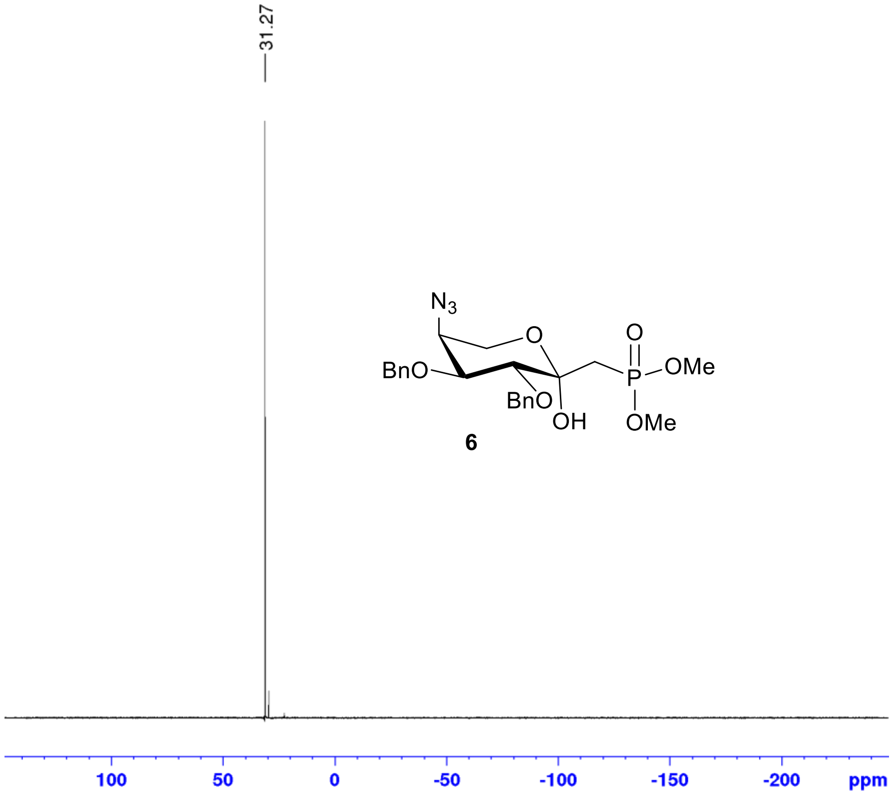

31.27
N3
O
BnO
BnO
OH
P
OMe
OMe
6
100
50
0
-50
-100
-150
-200
ppm

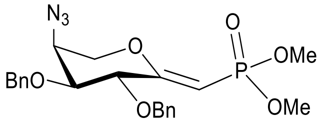

N3
O
BnO
OBn
O
P
OMe
OMe

8

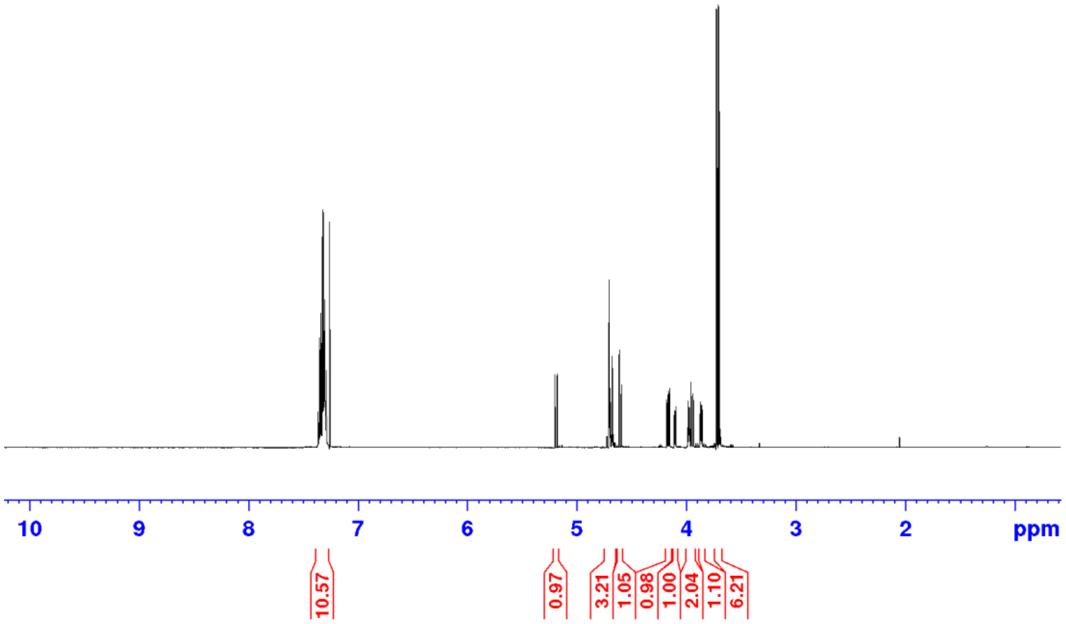

10
9
8
7
6
5
4
3
2
ppm
10.57
0.97
3.21
1.05
0.98
1.00
2.04
1.10
6.21

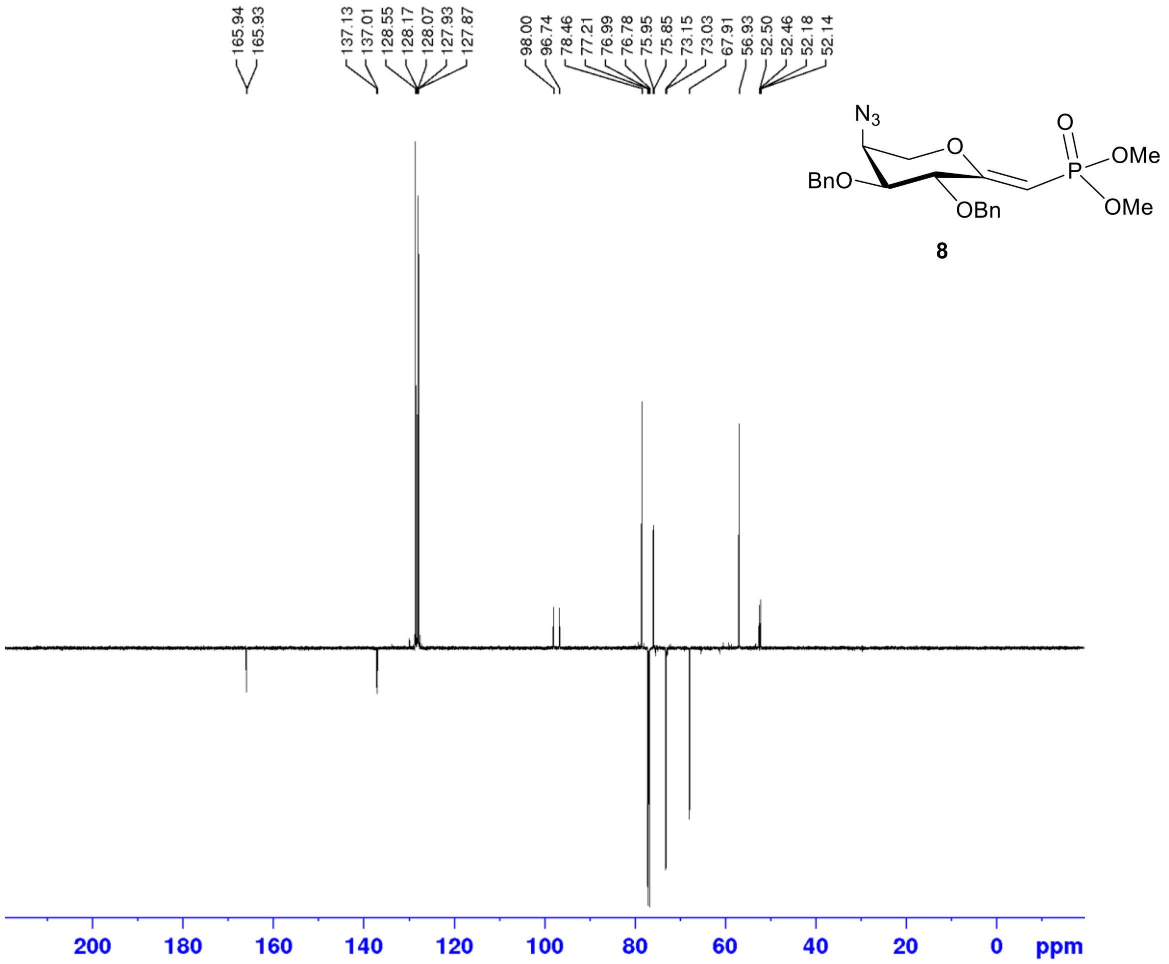
165.94
165.93
137.13
137.01
128.55
128.17
128.07
127.93
127.87
98.00
96.74
78.46
77.21
76.99
76.78
75.95
75.85
73.15
73.03
67.91
56.93
52.50
52.46
52.18
52.14
N3
O
BnO
OBn
P
OMe
OMe
8
200
180
160
140
120
100
80
60
40
20
0
ppm

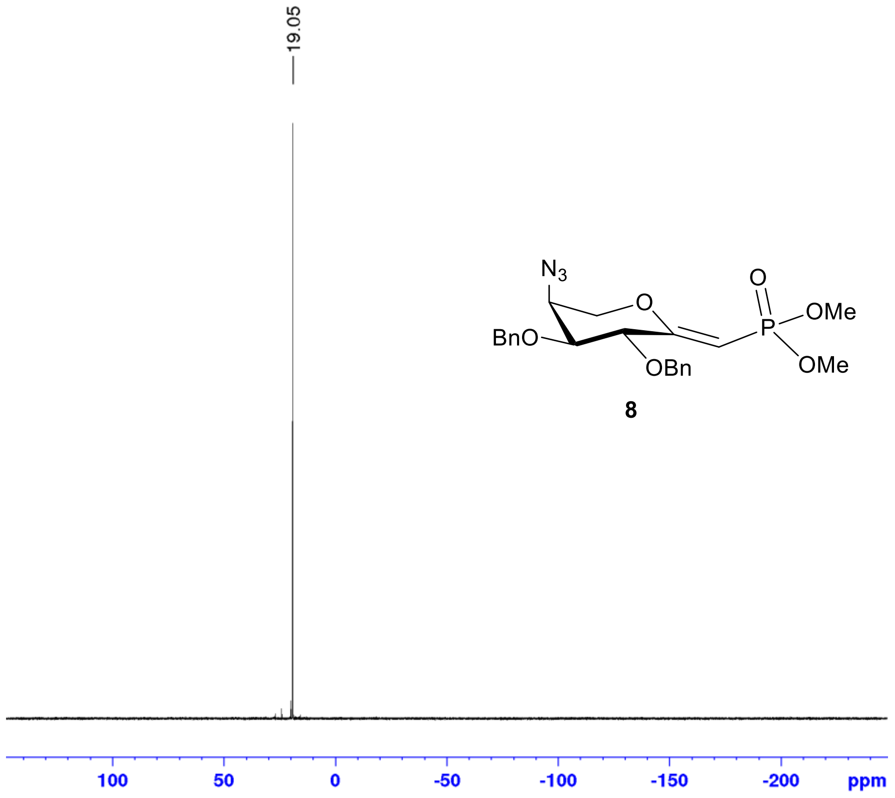
19.05
N3
O
BnO
OBn
P
OMe
OMe
8
100
50
0
-50
-100
-150
-200
ppm

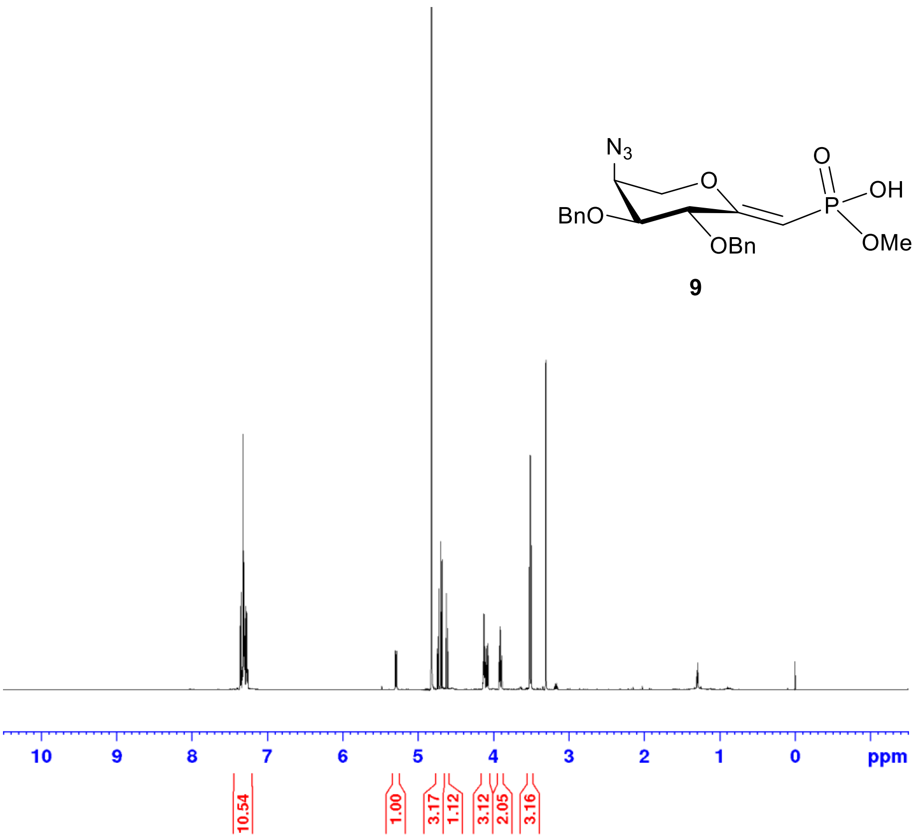
N3
BnO
O
OBn
O
P
OH
OMe
9
10
9
8
7
6
5
4
3
2
1
0
ppm
10.54
1.00
3.17
1.12
3.12
2.05
3.16

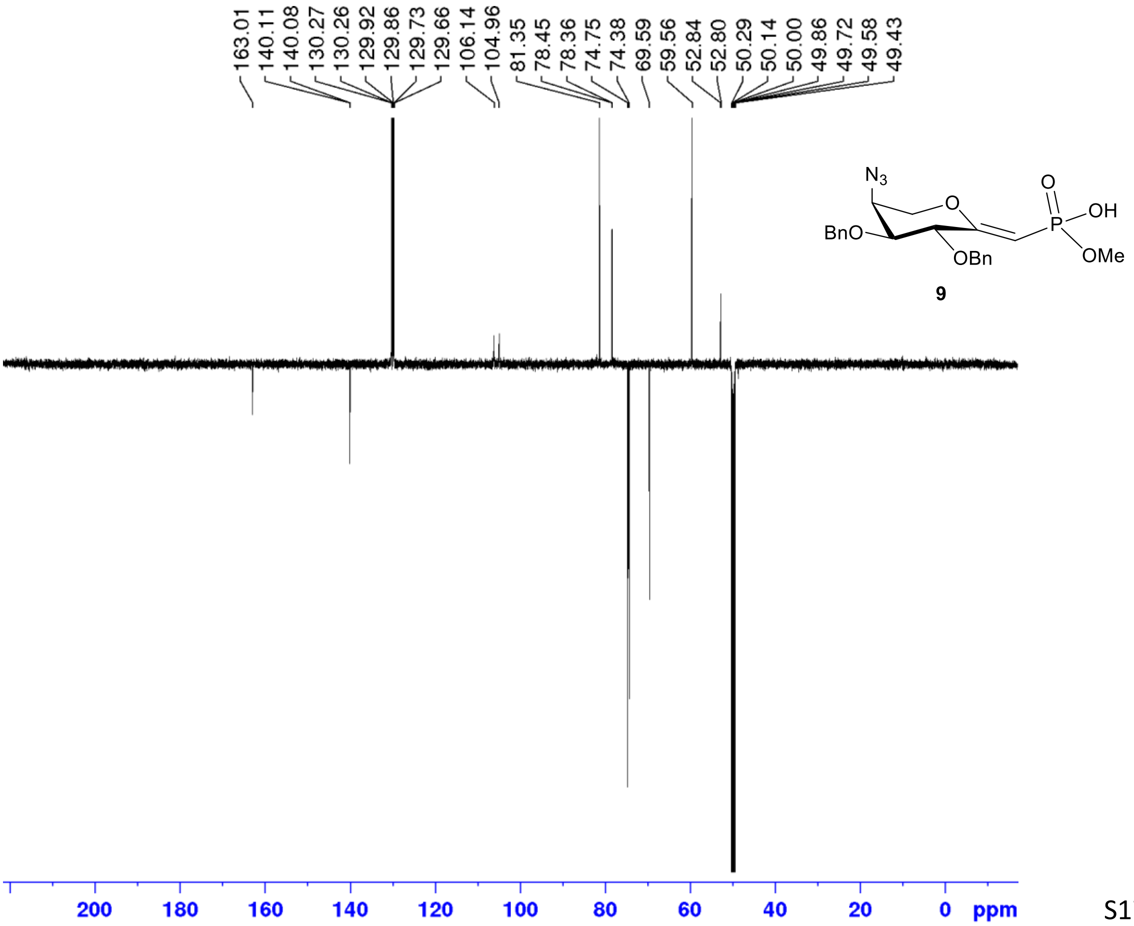
163.01
140.11
140.08
130.27
130.26
129.92
129.86
129.73
129.66
106.14
104.96
81.35
78.45
78.36
74.75
74.38
69.59
59.56
52.84
52.80
50.29
50.14
50.00
49.86
49.72
49.58
49.43
N3
BnO
O
OBn
O
P
OH
OMe
9
200
180
160
140
120
100
80
60
40
20
0
ppm

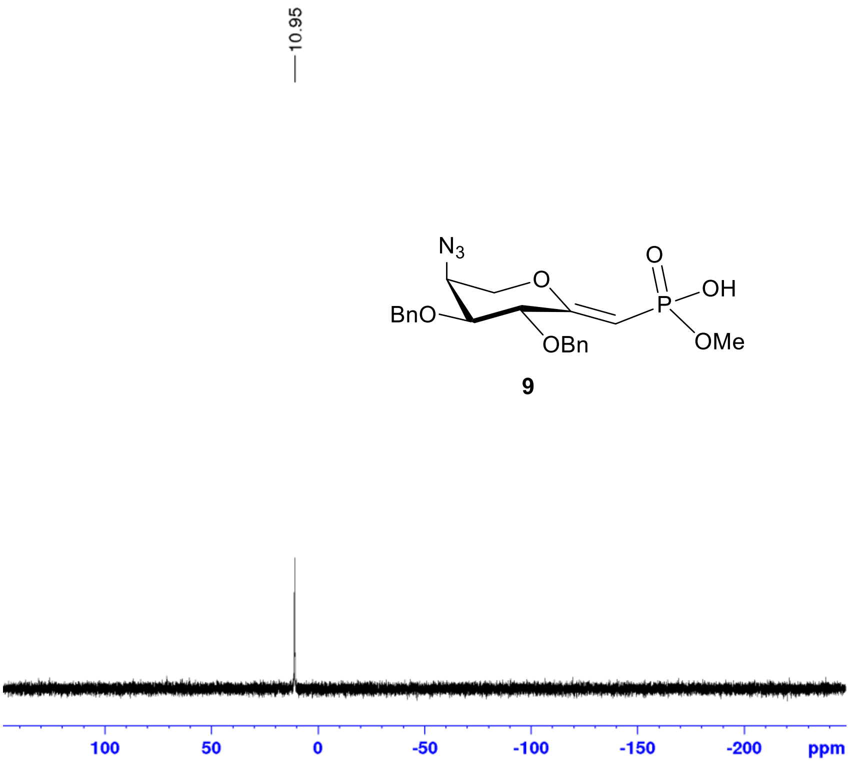
10.95
N3
O
O
OH
P
BnO
OBn
OMe
9
100
50
0
-50
-100
-150
-200
ppm

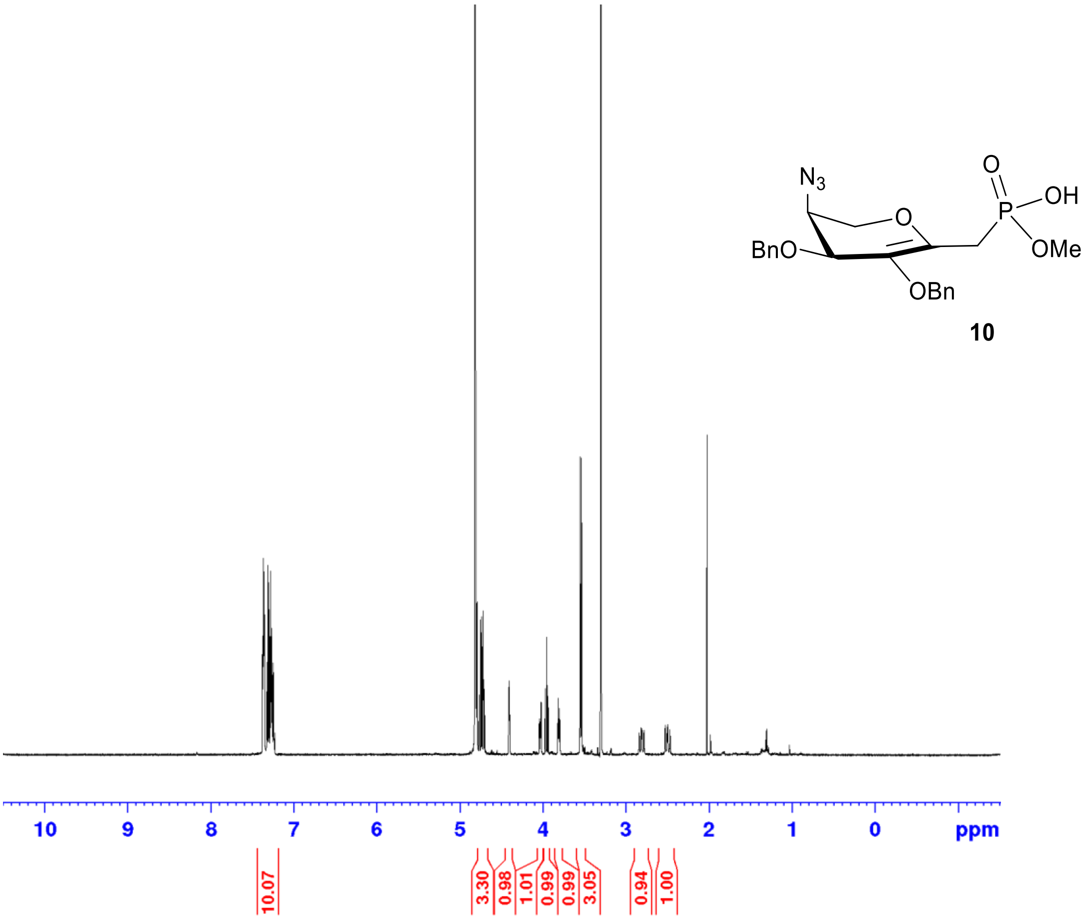
N3
O
O
OH
P
BnO
OMe
OBn
10
10
9
8
7
6
5
4
3
2
1
0
ppm
10.07
3.30
0.98
1.01
0.99
0.99
3.05
0.94
1.00

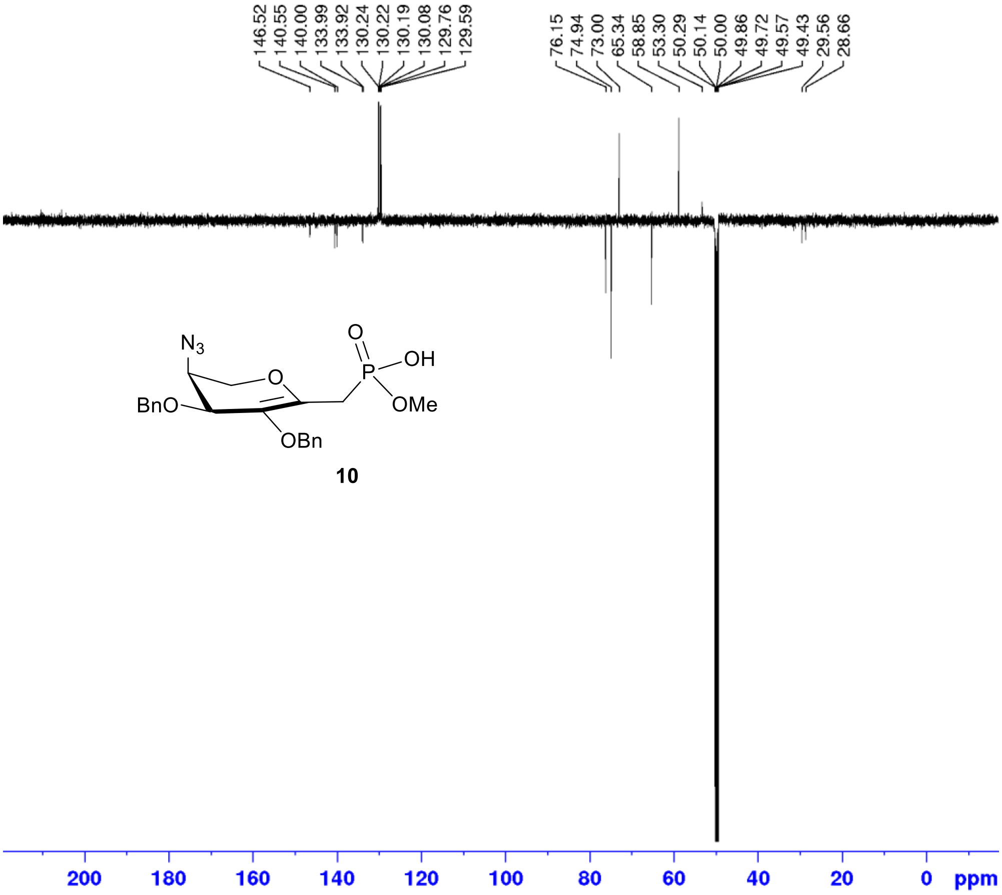

146.52
140.55
140.00
133.99
133.92
130.24
130.22
130.19
130.08
129.76
129.59
76.15
74.94
73.00
65.34
58.85
53.30
50.29
50.14
50.00
49.86
49.72
49.57
49.43
29.56
28.66
N3
O
O
P
OH
BnO
OMe
OBn
10
200
180
160
140
120
100
80
60
40
20
0
ppm

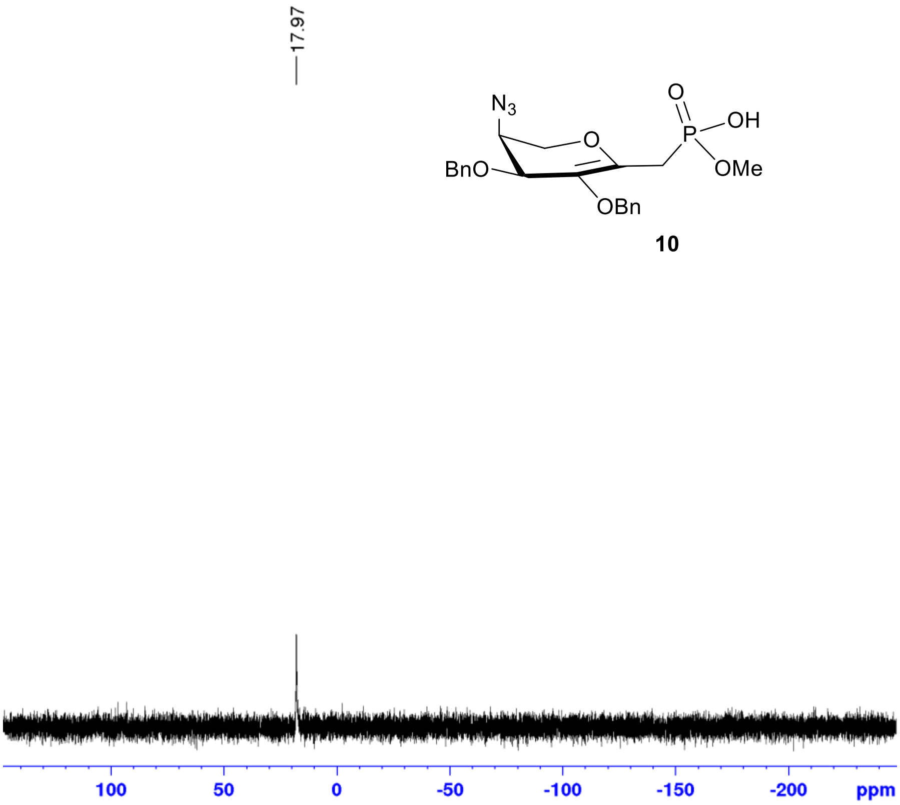

17.97
N3
O
O
P
OH
BnO
OMe
OBn
10
100
50
0
-50
-100
-150
-200
ppm

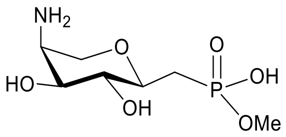

11

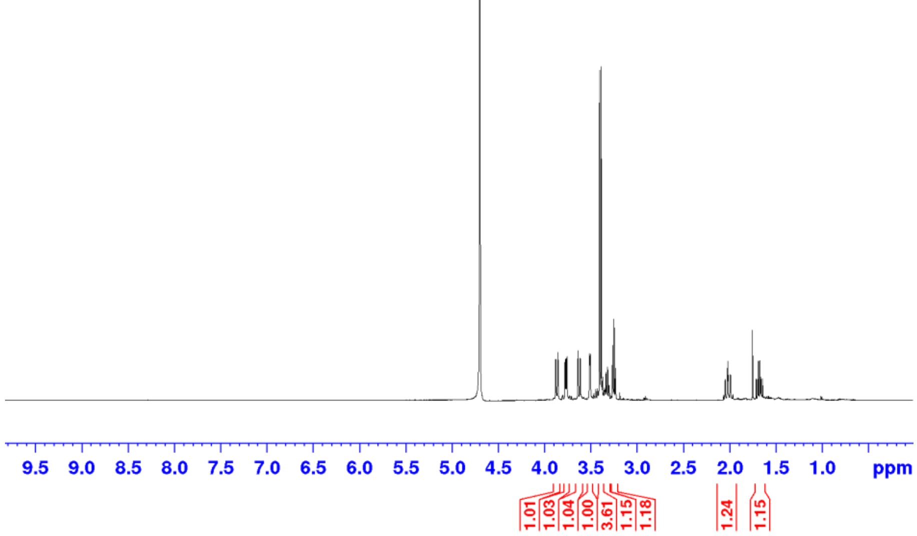
9.5 9.0 8.5 8.0 7.5 7.0 6.5 6.0 5.5 5.0 4.5 4.0 3.5 3.0 2.5 2.0 1.5 1.0 ppm
1.01 1.03 1.04 1.00 3.61 1.15 1.18 1.24 1.15

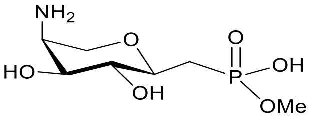

11

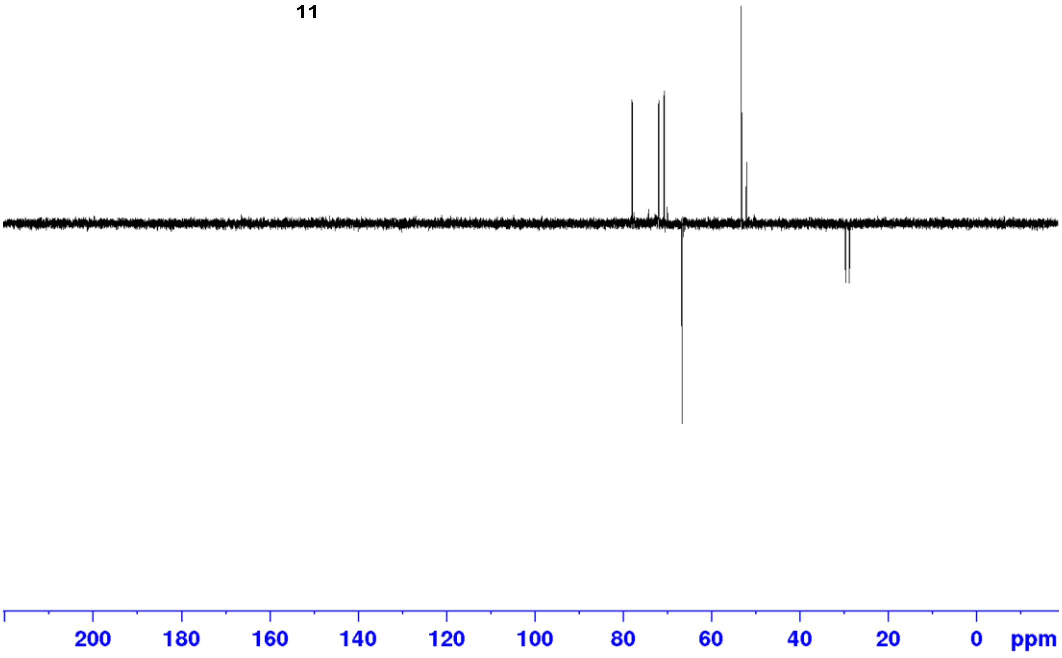
77.94 77.90 72.01 71.92 70.69 66.62 53.19 52.06 52.02 29.62 28.72
200 180 160 140 120 100 80 60 40 20 0 ppm

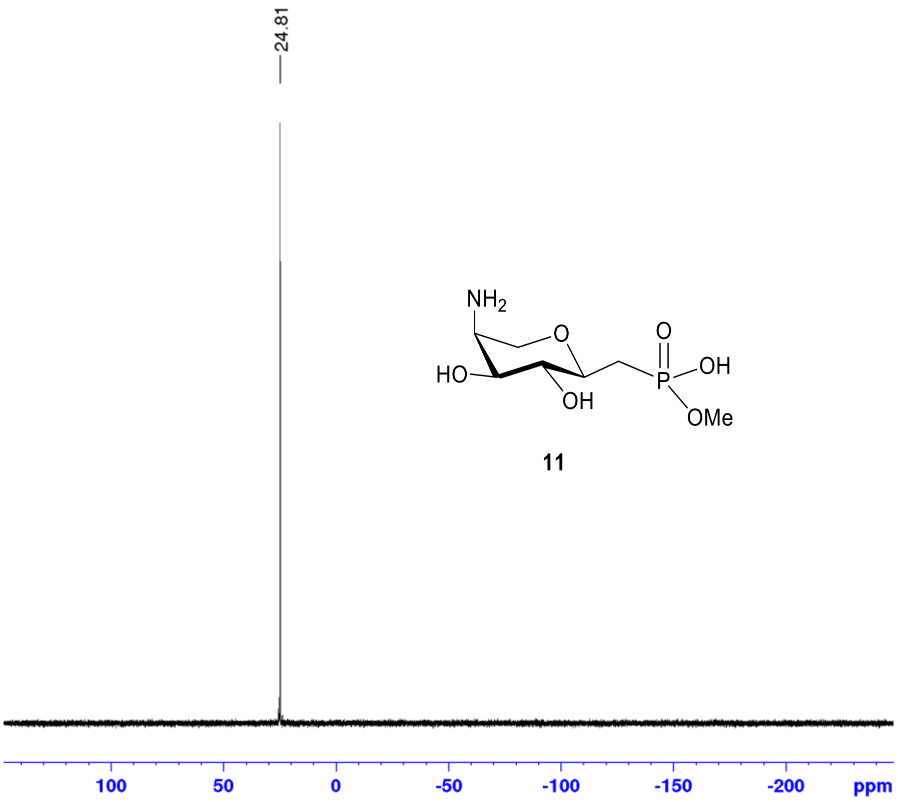
24.81
NH2
O
HO
OH
P
OH
OMe
11
100
50
0
-50
-100
-150
-200
ppm

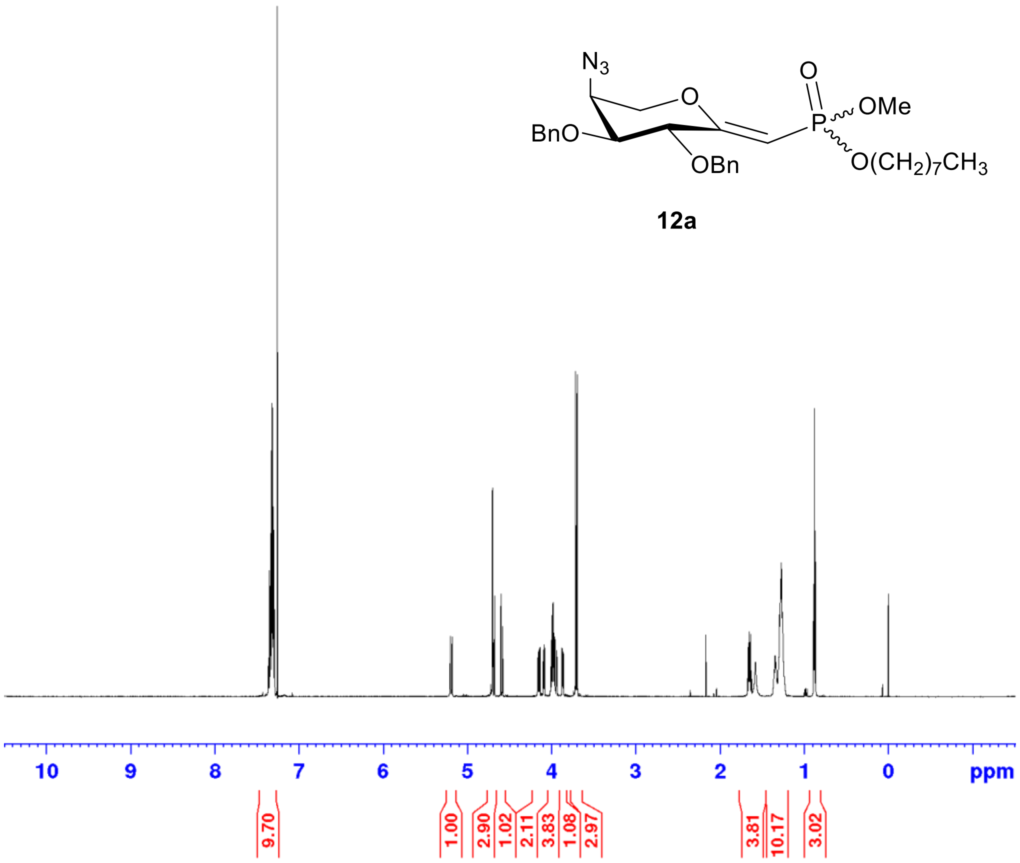
N3
O
BnO
OBn
P
OMe
O(CH2)7CH3
12a
10
9
8
7
6
5
4
3
2
1
0
ppm
9.70
1.00
2.90
1.02
2.11
3.83
1.08
2.97
3.81
10.17
3.02

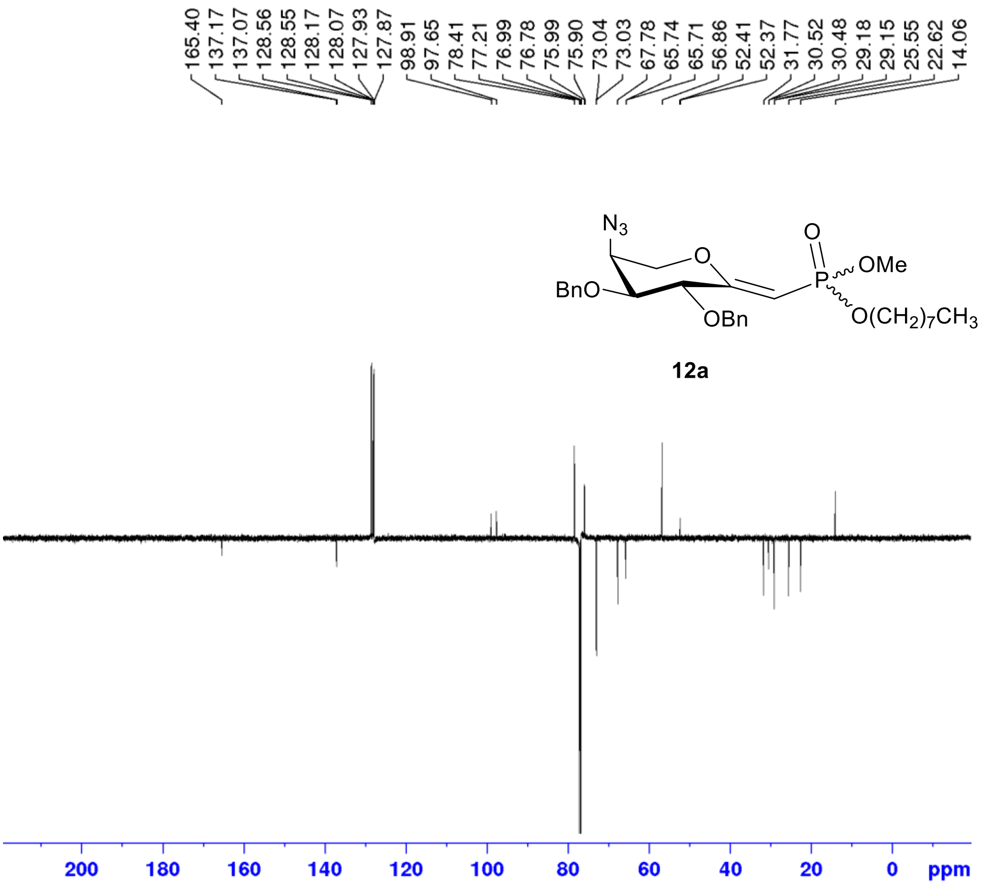
165.40
137.17
137.07
128.56
128.55
128.17
128.07
127.93
127.87
98.91
97.65
78.41
77.21
76.99
76.78
75.99
75.90
73.04
73.03
67.78
65.74
65.71
56.86
52.41
52.37
31.77
30.52
30.48
29.18
29.15
25.55
22.62
14.06
N_3
O
BnO
OBn
O
P
OMe
$O(CH_2)_7CH_3$
12a
200 180 160 140 120 100 80 60 40 20 0 ppm

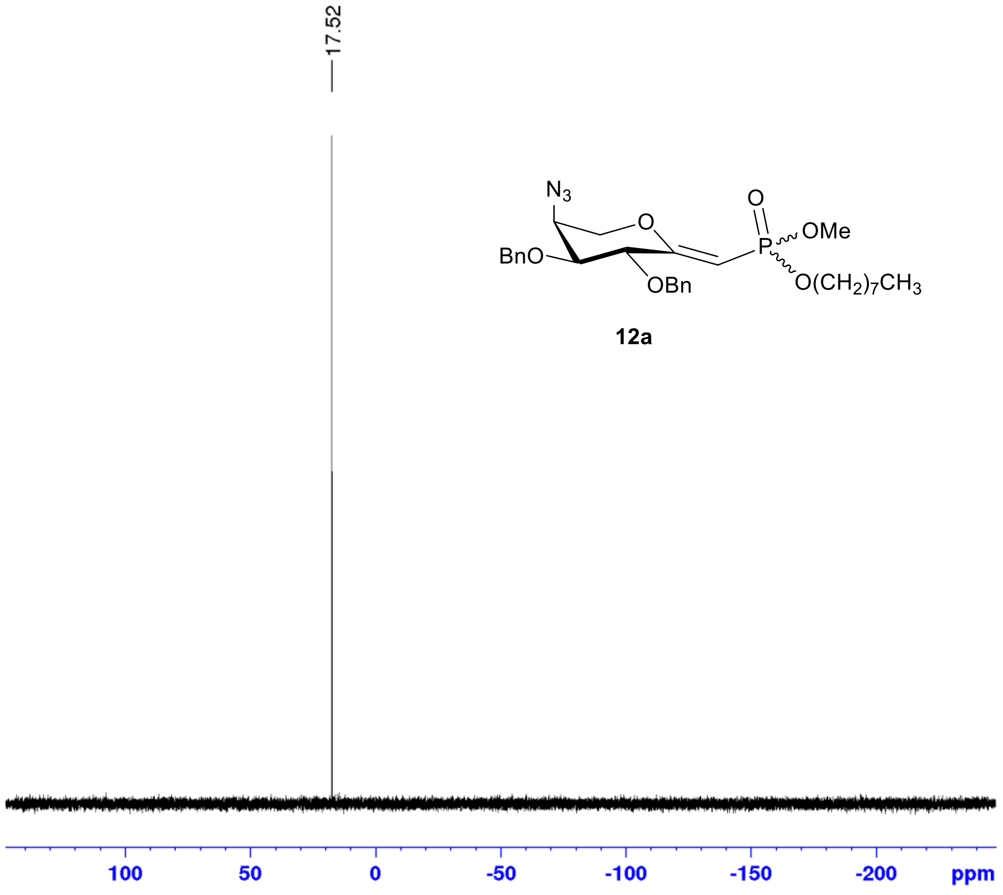
17.52
N_3
O
BnO
OBn
O
P
OMe
$O(CH_2)_7CH_3$
12a
100 50 0 -50 -100 -150 -200 ppm

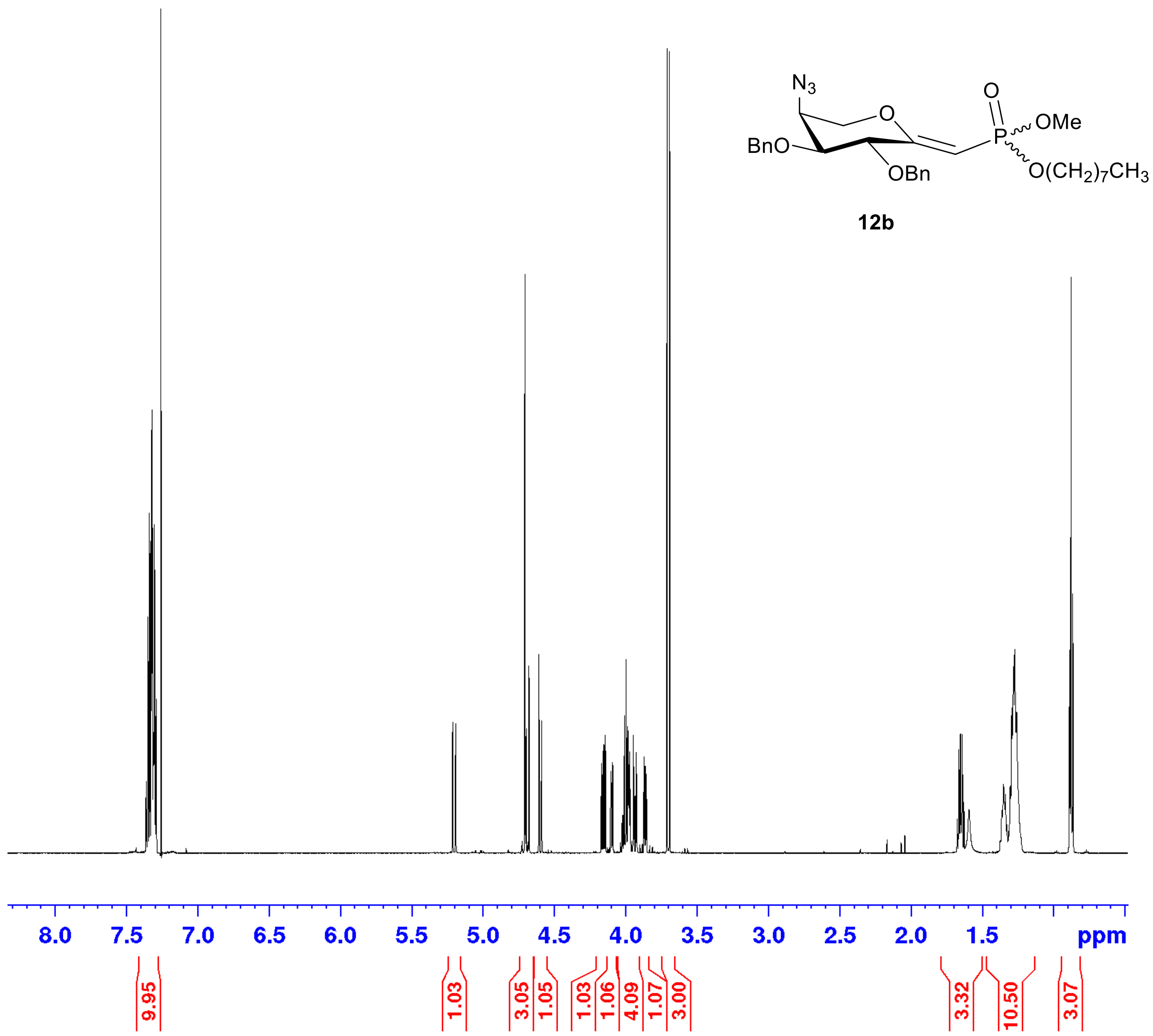
N3
O
BnO
OBn
O
P
OMe
O(CH2)7CH3
12b
8.0 7.5 7.0 6.5 6.0 5.5 5.0 4.5 4.0 3.5 3.0 2.5 2.0 1.5 ppm
9.95
1.03
3.05
1.05
1.03
1.06
4.09
1.07
3.00
3.32
10.50
3.07

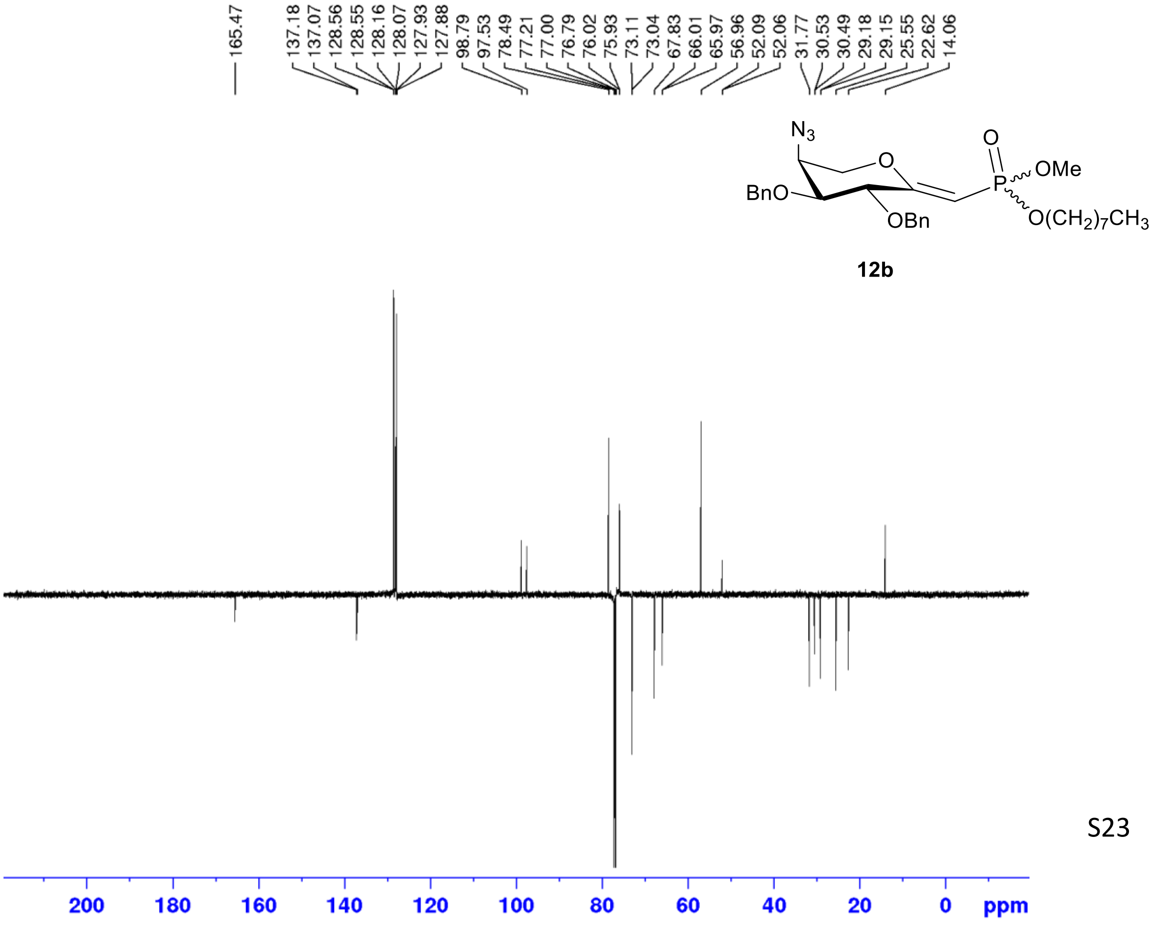
165.47
137.18
137.07
128.56
128.55
128.16
128.07
127.93
127.88
98.79
97.53
78.49
77.21
77.00
76.79
76.02
75.93
73.11
73.04
67.83
66.01
65.97
56.96
52.09
52.06
31.77
30.53
30.49
29.18
29.15
25.55
22.62
14.06
N3
O
BnO
OBn
O
P
OMe
O(CH2)7CH3
12b
200 180 160 140 120 100 80 60 40 20 0 ppm

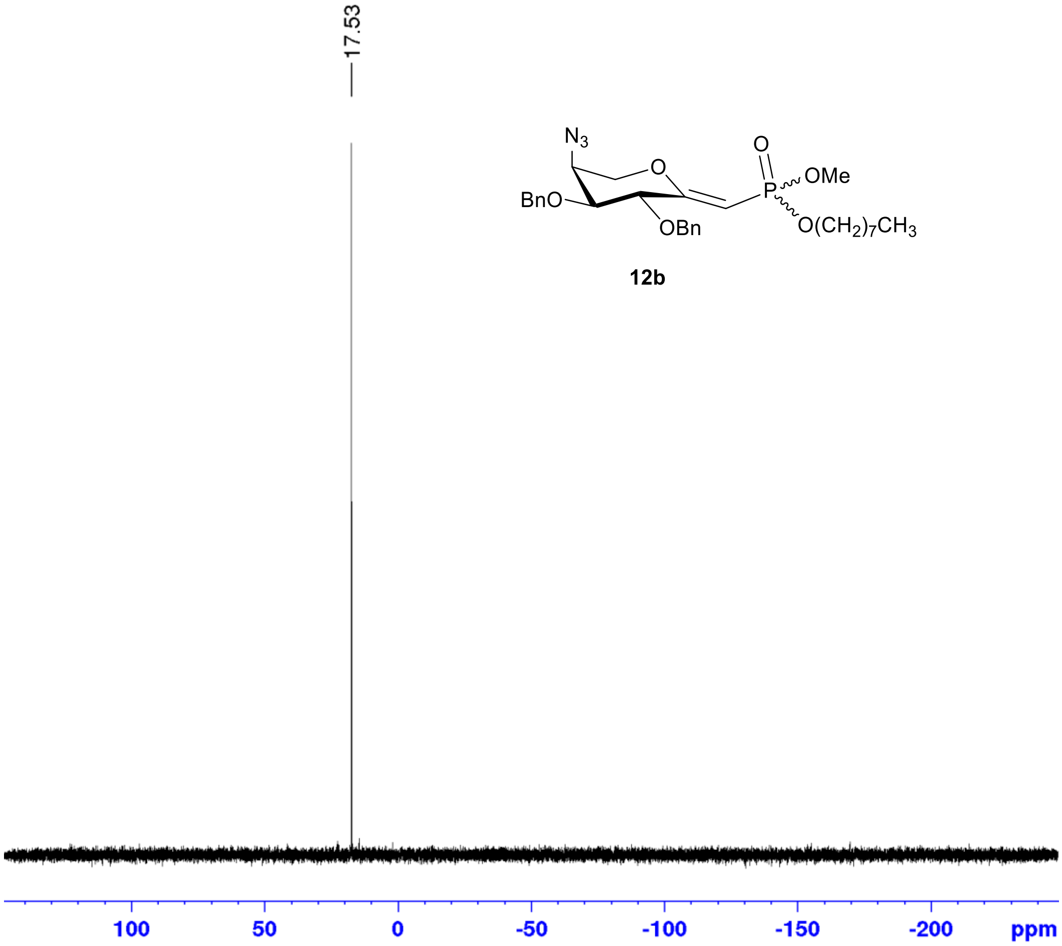

17.53
100
50
0
-50
-100
-150
-200
ppm
N3
O
O
P
OMe
BnO
OBn
O(CH2)7CH3

12b

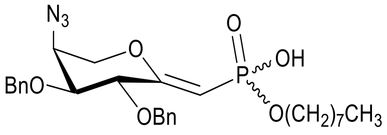

N3
O
O
P
OH
BnO
OBn
O(CH2)7CH3

13

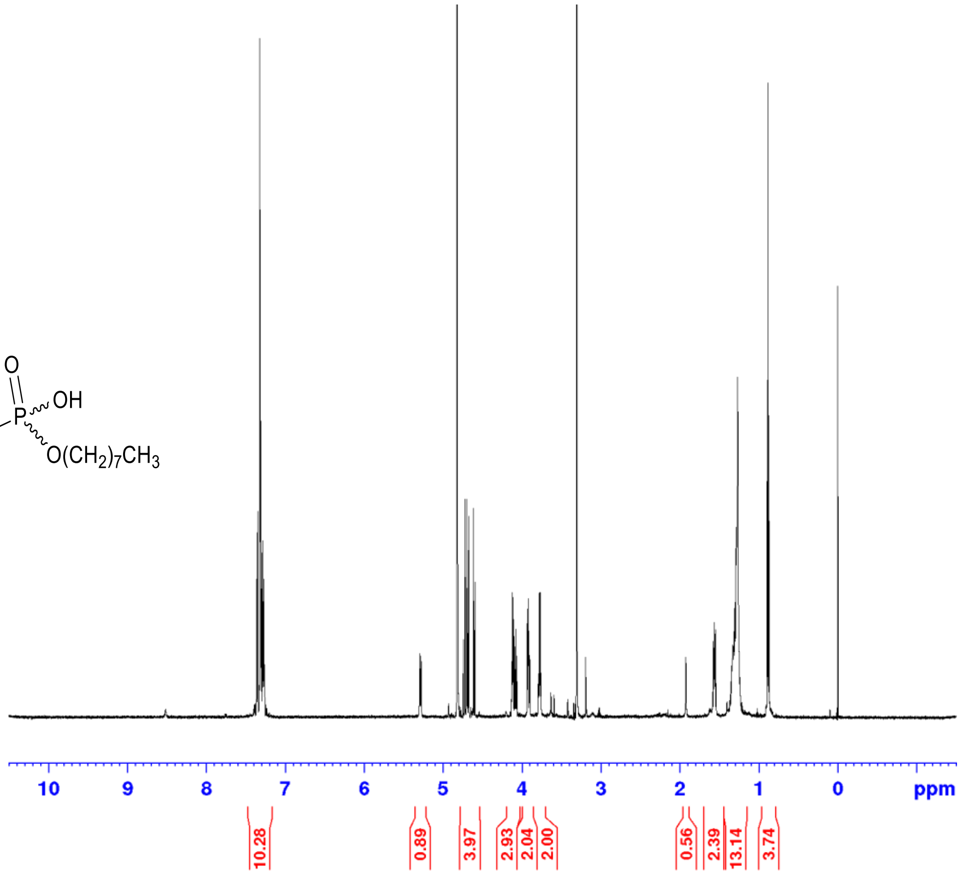

10
9
8
7
6
5
4
3
2
1
0
ppm
10.28
0.89
3.97
2.93
2.04
2.00
0.56
2.39
13.14
3.74

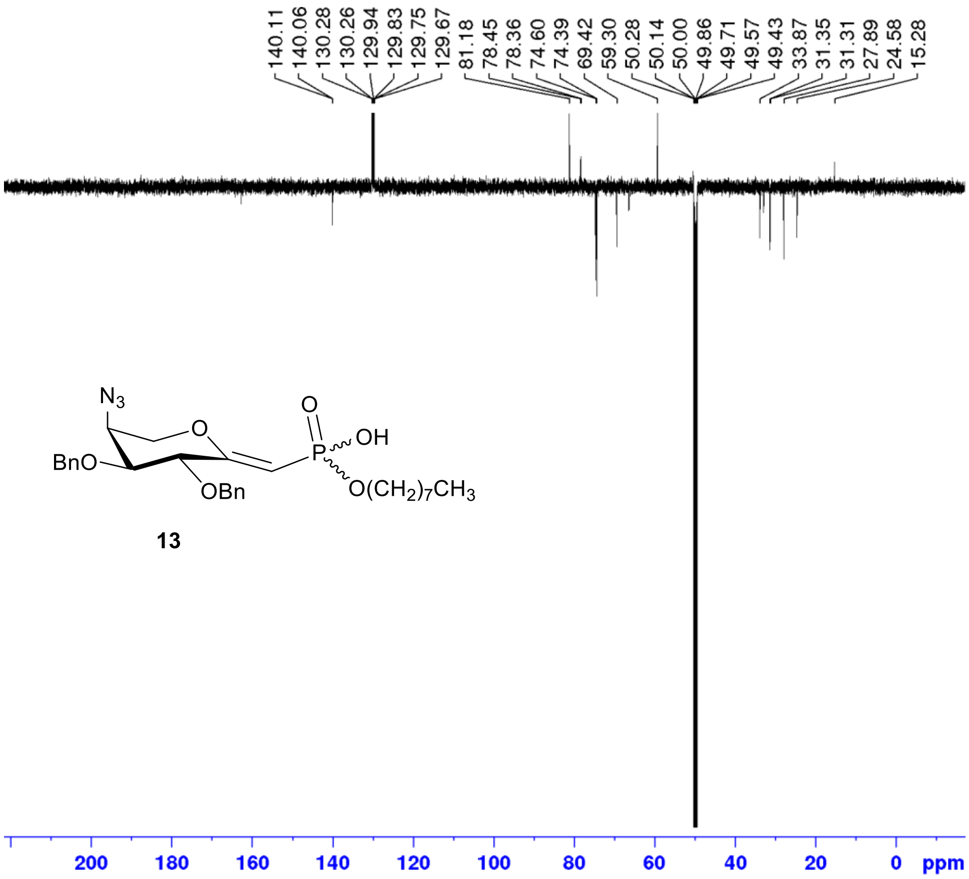
140.11
140.06
130.28
130.26
129.94
129.83
129.75
129.67
81.18
78.45
78.36
74.60
74.39
69.42
59.30
50.28
50.14
50.00
49.86
49.71
49.57
49.43
33.87
31.35
31.31
27.89
24.58
15.28
N3
O
BnO
OBn
O
P
OH
O(CH2)7CH3
200 180 160 140 120 100 80 60 40 20 0 ppm

13

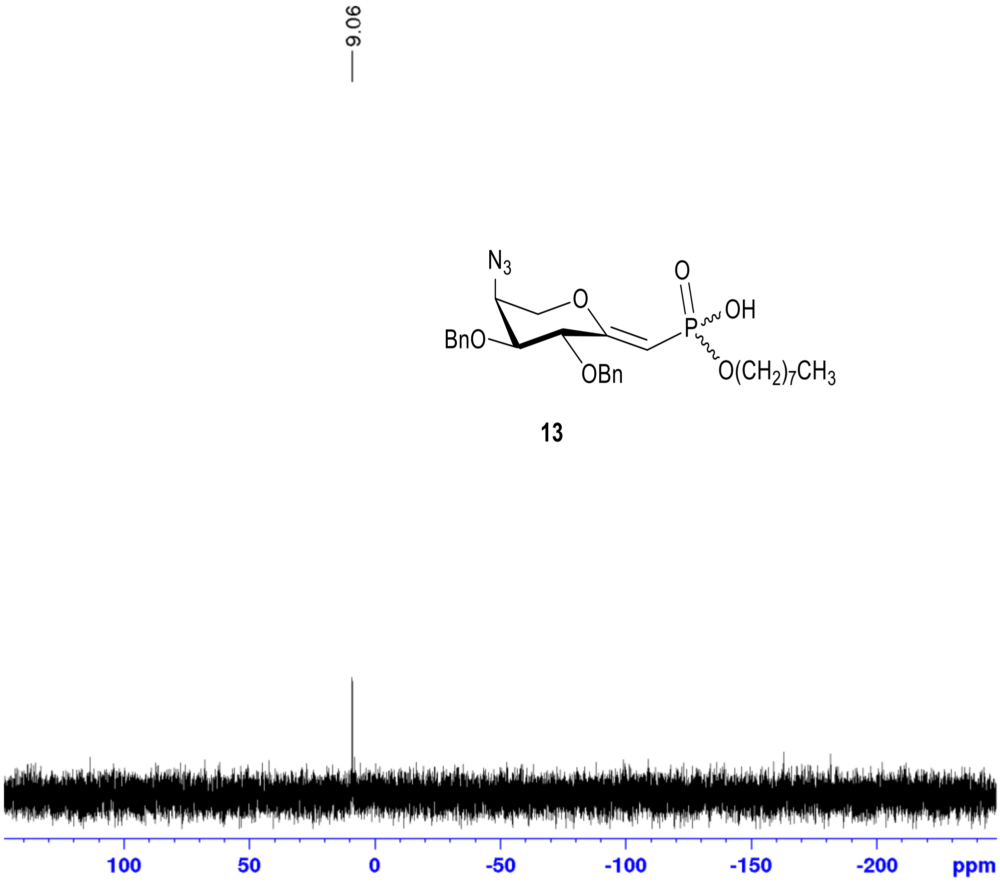
9.06
N3
O
BnO
OBn
O
P
OH
O(CH2)7CH3
100 50 0 -50 -100 -150 -200 ppm

13

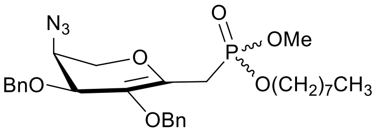
N3
O
BnO
OBn
O
P
OMe
O(CH2)7CH3

14a

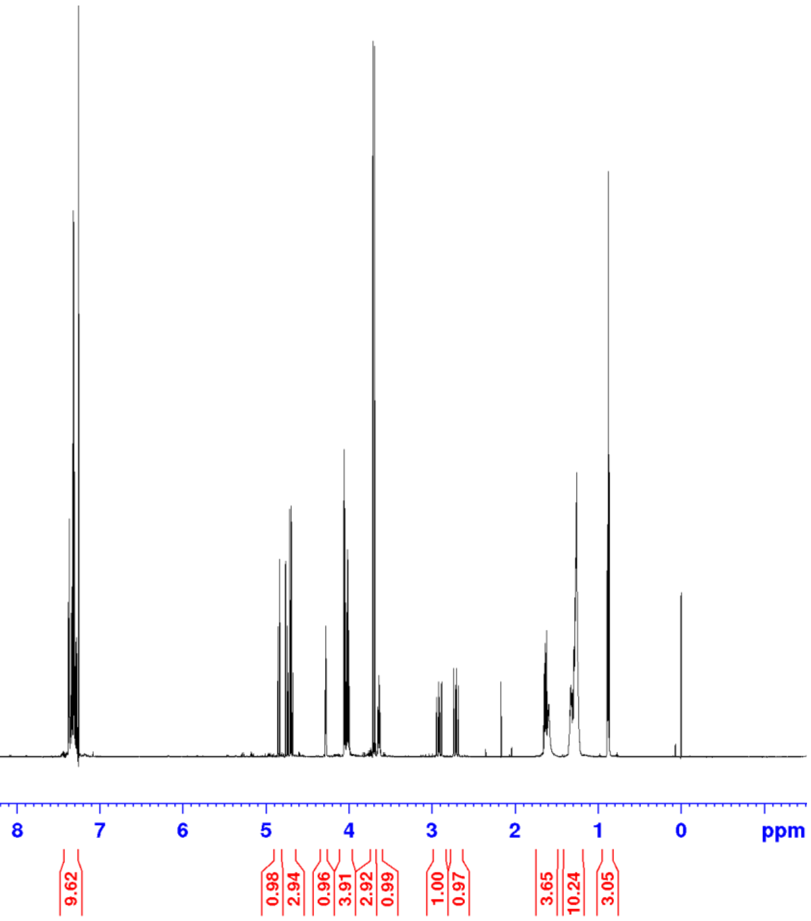
10 9 8 7 6 5 4 3 2 1 0 ppm
9.62
0.98 2.94 0.96 3.91 2.92 0.99
1.00 0.97
3.65 10.24 3.05

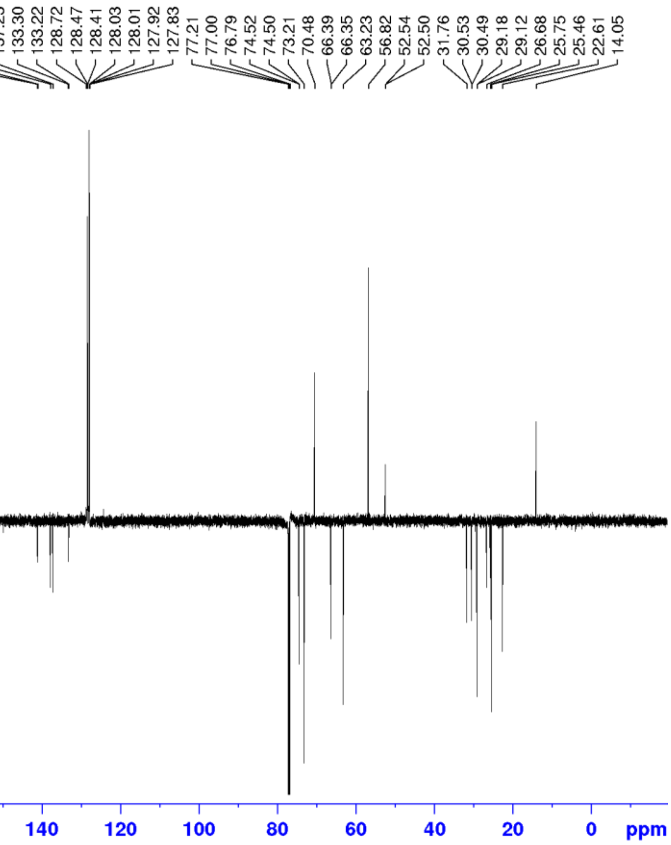
141.20 141.11 137.86 137.23 133.30 133.22 128.72 128.47 128.41 128.03 128.01 127.92 127.83 77.21 77.00 76.79 74.52 74.50 73.21 70.48 66.39 66.35 63.23 56.82 52.54 52.50 31.76 30.53 30.49 29.18 29.12 26.68 25.75 25.46 22.61 14.05
200 180 160 140 120 100 80 60 40 20 0 ppm

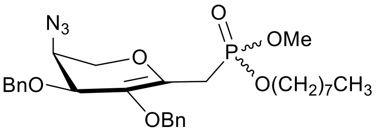
N3
O
BnO
OBn
O
P
OMe
O(CH2)7CH3

14a

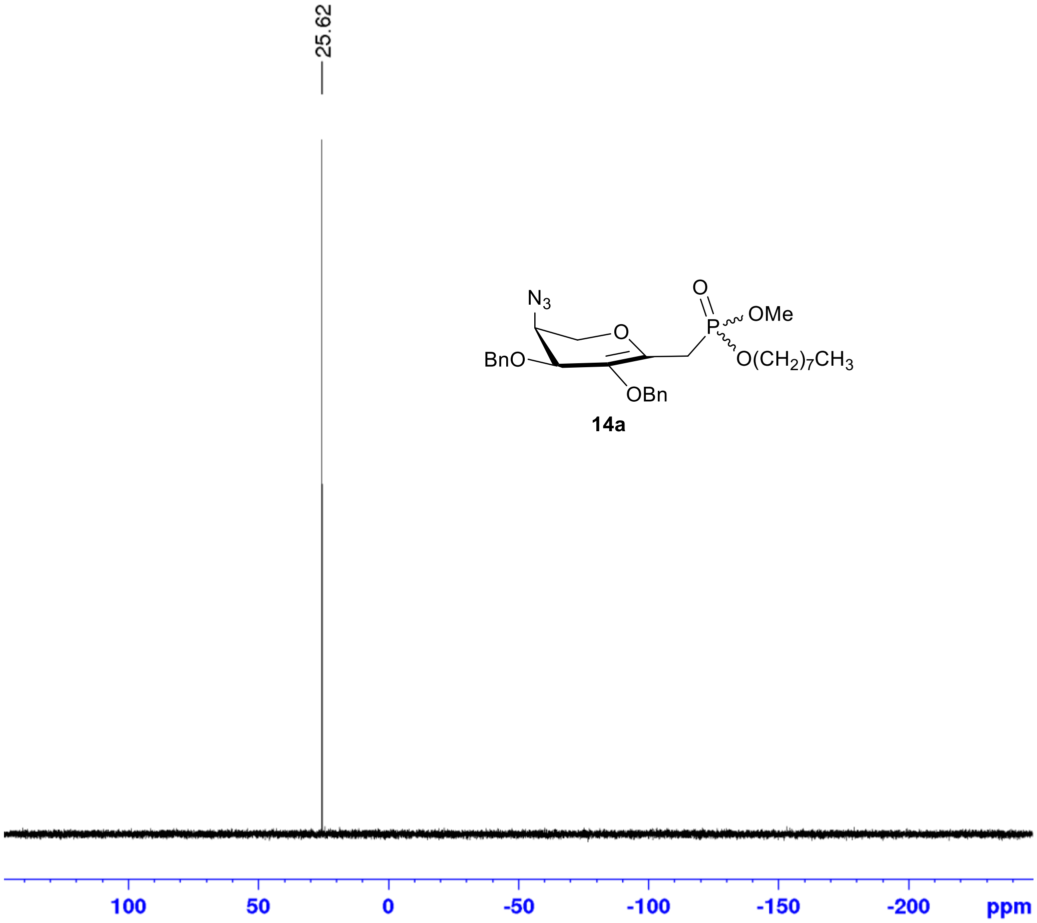
25.62
N3
O
O
P
OMe
O(CH2)7CH3
BnO
OBn
14a
100
50
0
-50
-100
-150
-200
ppm

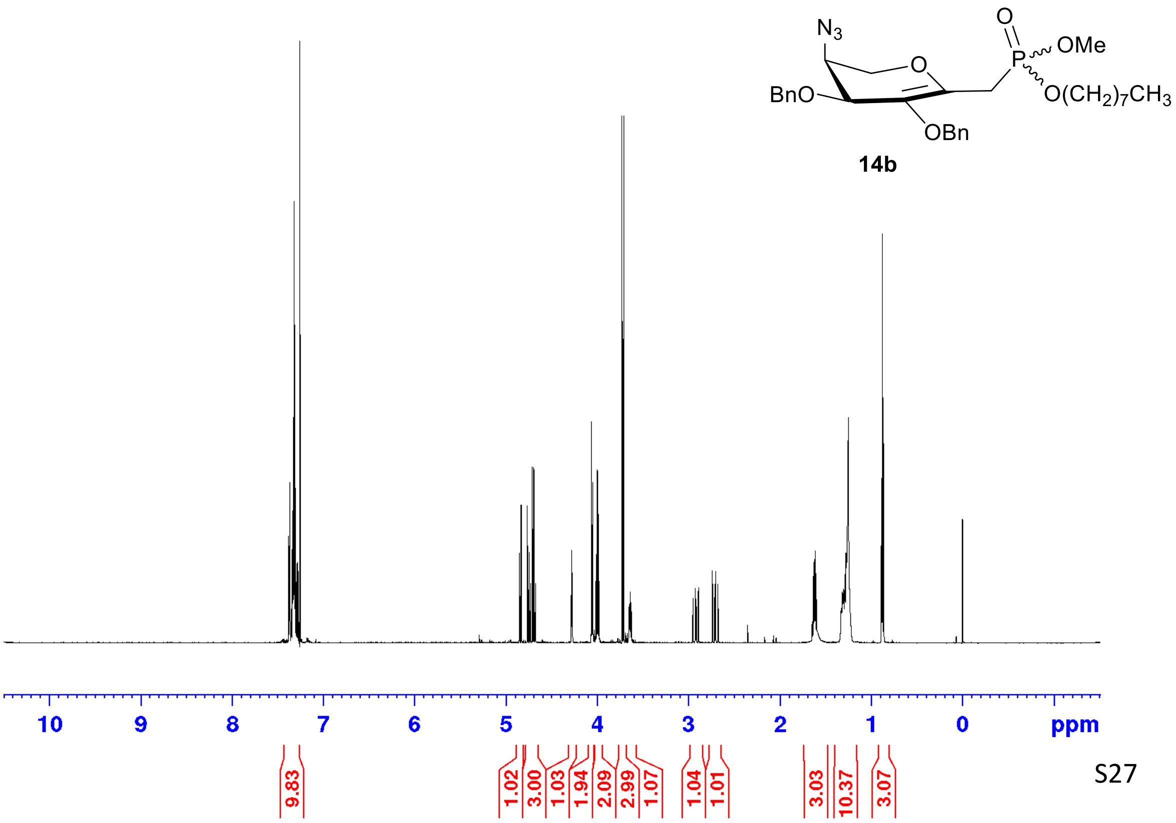
N3
O
O
P
OMe
O(CH2)7CH3
BnO
OBn
14b
10
9
8
7
6
5
4
3
2
1
0
ppm
9.83
1.02
3.00
1.03
1.94
2.09
2.99
1.07
1.04
1.01
3.03
10.37
3.07

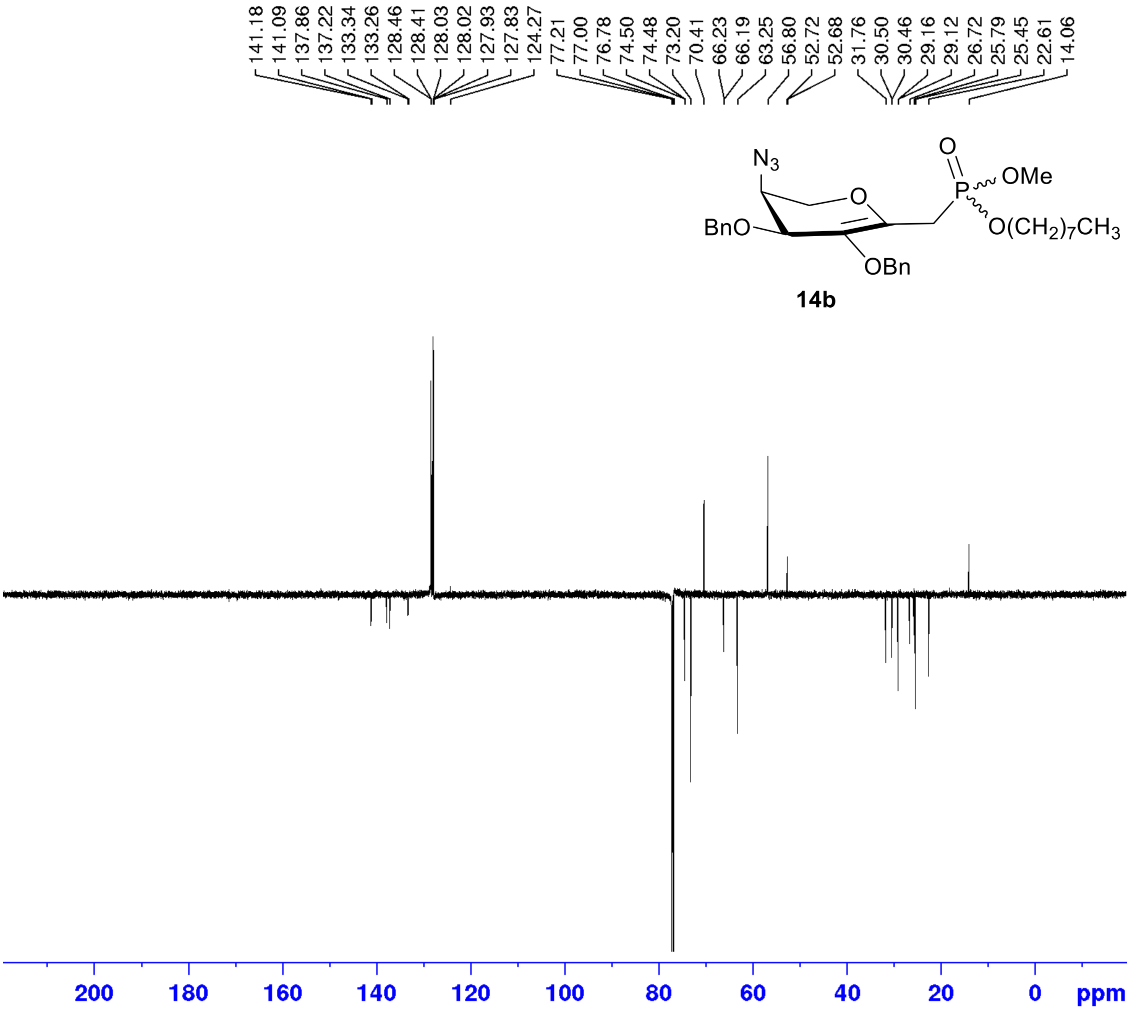
141.18
141.09
137.86
137.22
133.34
133.26
128.46
128.41
128.03
128.02
127.93
127.83
124.27
77.21
77.00
76.78
74.50
74.48
73.20
70.41
66.23
66.19
63.25
56.80
52.72
52.68
31.76
30.50
30.46
29.16
29.12
26.72
25.79
25.45
22.61
14.06
N3
O
P
OMe
BnO
O(CH2)7CH3
OBn
14b
200
180
160
140
120
100
80
60
40
20
0
ppm

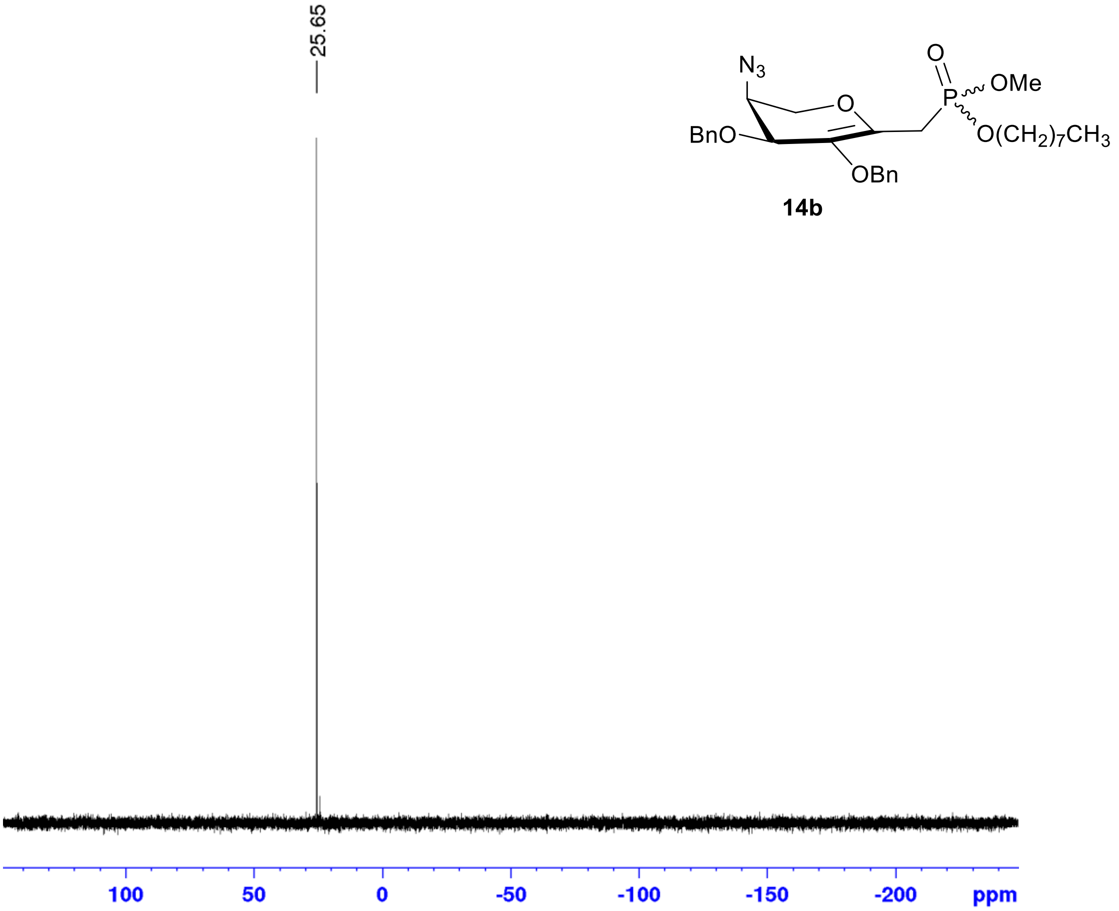
25.65
N3
O
P
OMe
BnO
O(CH2)7CH3
OBn
14b
100
50
0
-50
-100
-150
-200
ppm

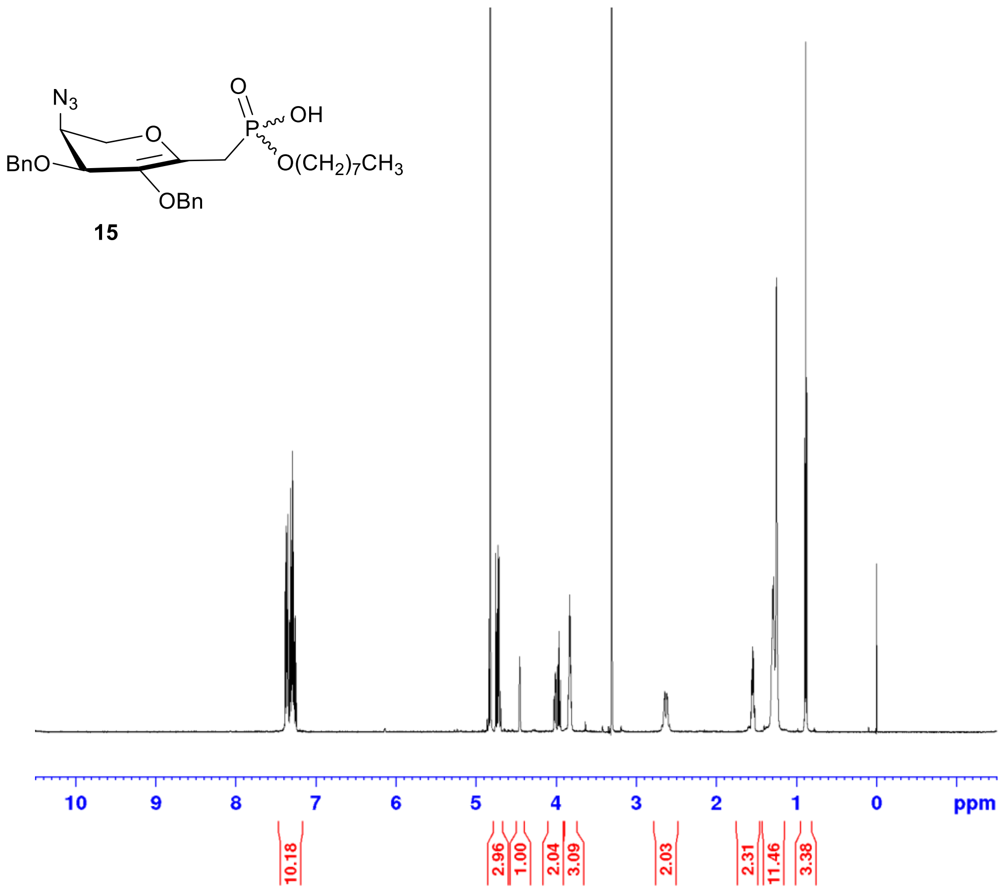
N3
O
P
OH
BnO
O(CH2)7CH3
OBn
15
10 9 8 7 6 5 4 3 2 1 0 ppm
10.18
2.96
1.00
2.04
3.09
2.03
2.31
11.46
3.38

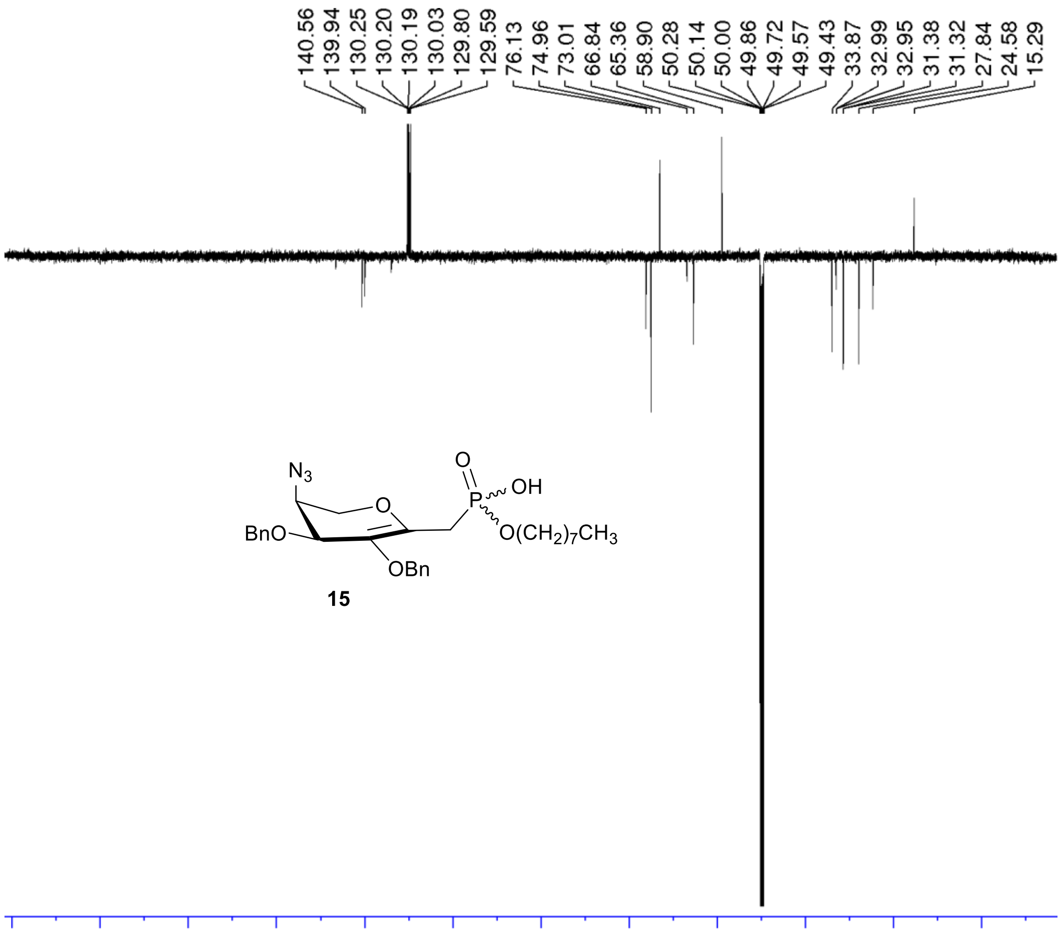
140.56
139.94
130.25
130.20
130.19
130.03
129.80
129.59
76.13
74.96
73.01
66.84
65.36
58.90
50.28
50.14
50.00
49.86
49.72
49.57
49.43
33.87
32.99
32.95
31.38
31.32
27.84
24.58
15.29
N3
O
P
OH
BnO
O(CH2)7CH3
OBn
15
200 180 160 140 120 100 80 60 40 20 0 ppm

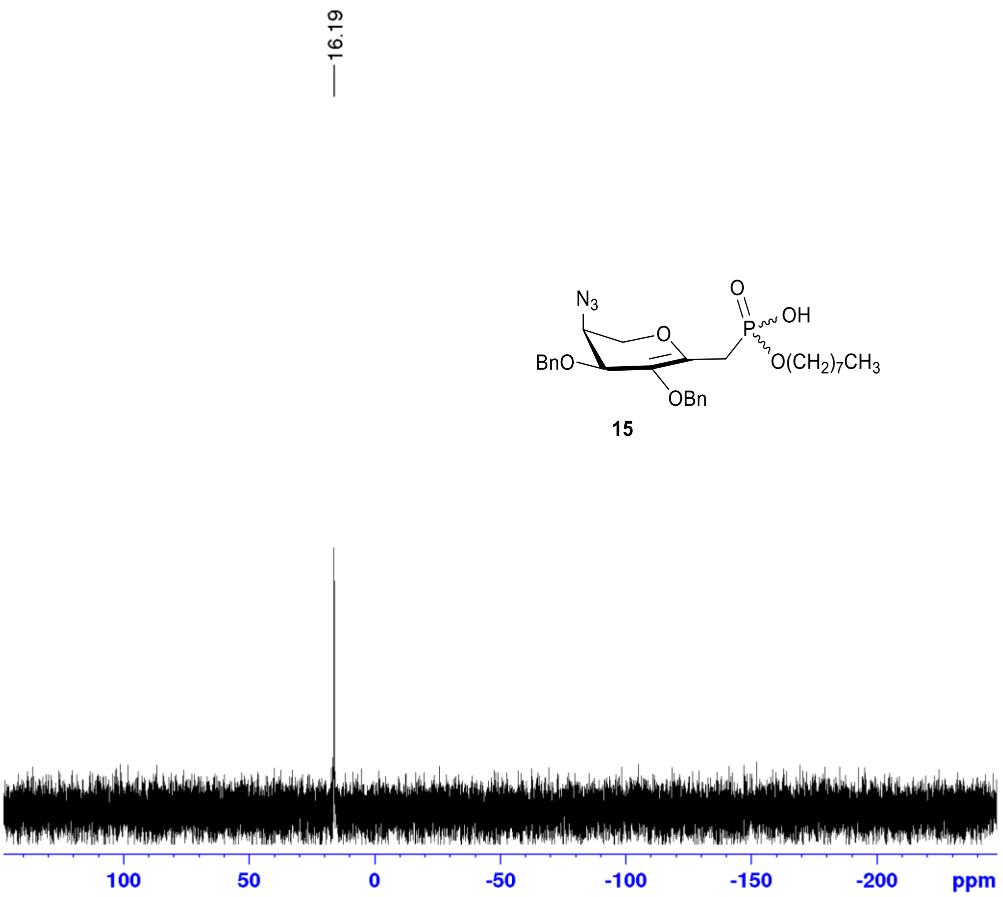

16.19
N3
O
BnO
OBn
O
P
OH
O(CH2)7CH3
100
50
0
-50
-100
-150
-200
ppm

15

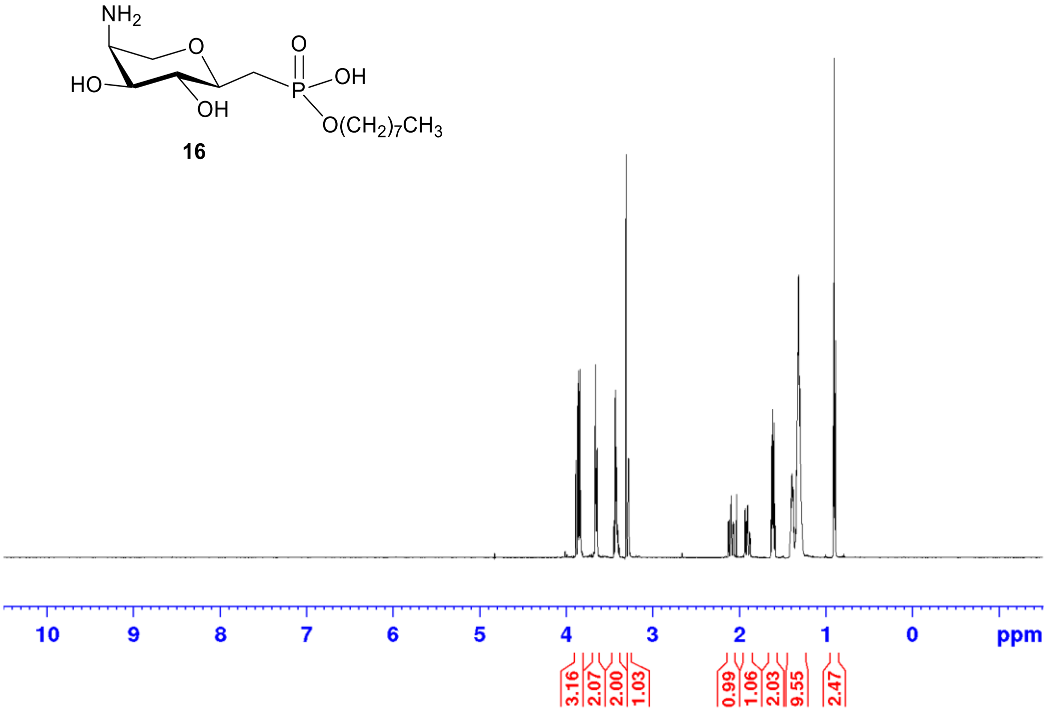

NH2
O
HO
OH
O
P
OH
O(CH2)7CH3
10
9
8
7
6
5
4
3
2
1
0
ppm
3.16
2.07
2.00
1.03
0.99
1.06
2.03
9.55
2.47

16

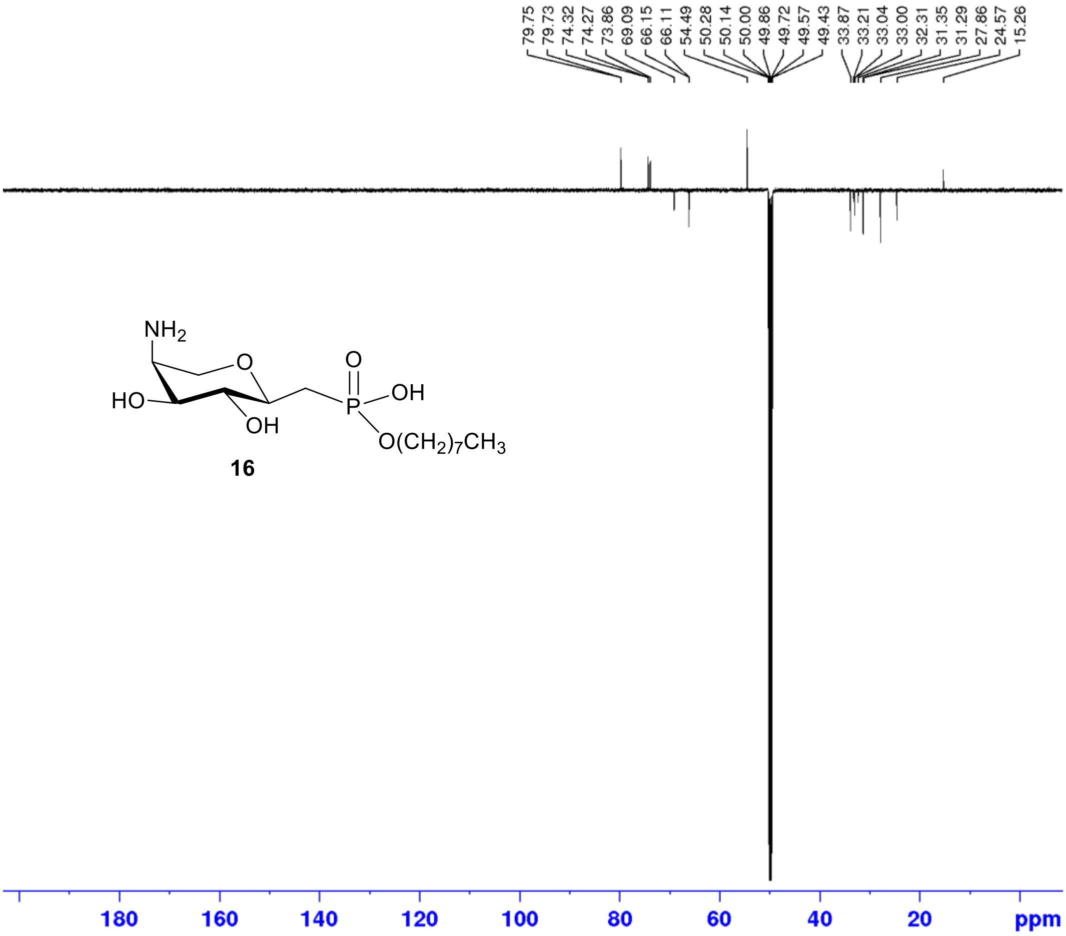
79.75
79.73
74.32
74.27
73.86
69.09
66.15
66.11
54.49
50.28
50.14
50.00
49.86
49.72
49.57
49.43
33.87
33.21
33.04
33.00
32.31
31.35
31.29
27.86
24.57
15.26
NH2
HO
OH
O
OH
O(CH2)7CH3
16
180
160
140
120
100
80
60
40
20
ppm

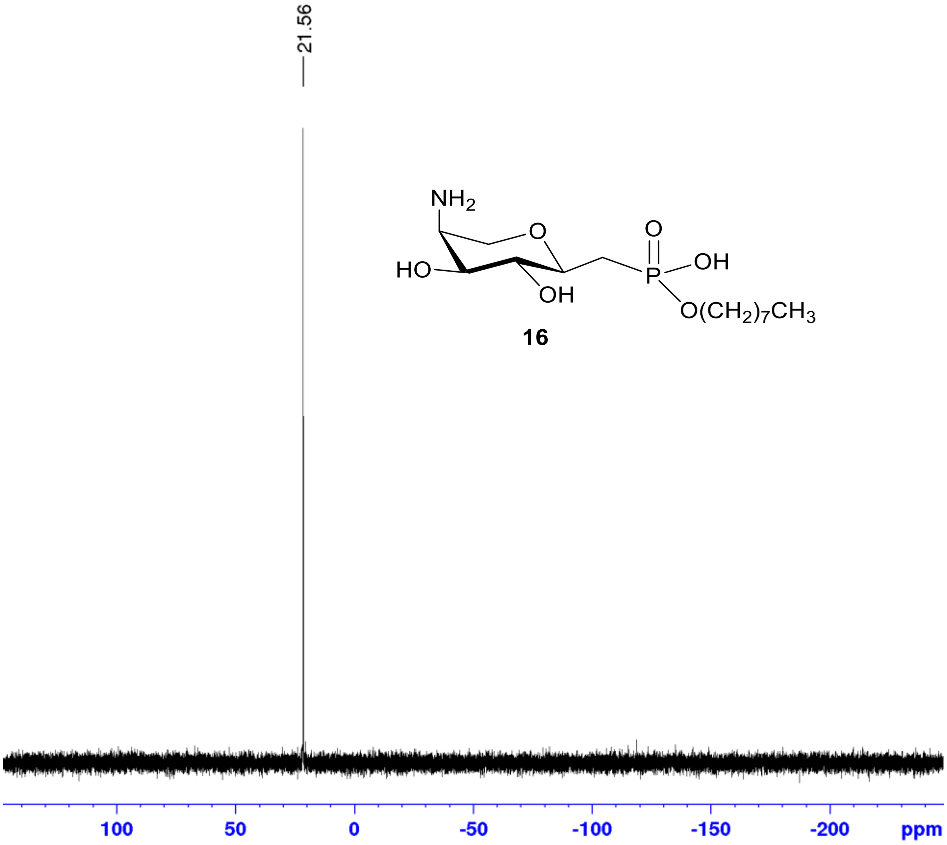
21.56
NH2
HO
OH
O
OH
O(CH2)7CH3
16
100
50
0
-50
-100
-150
-200
ppm

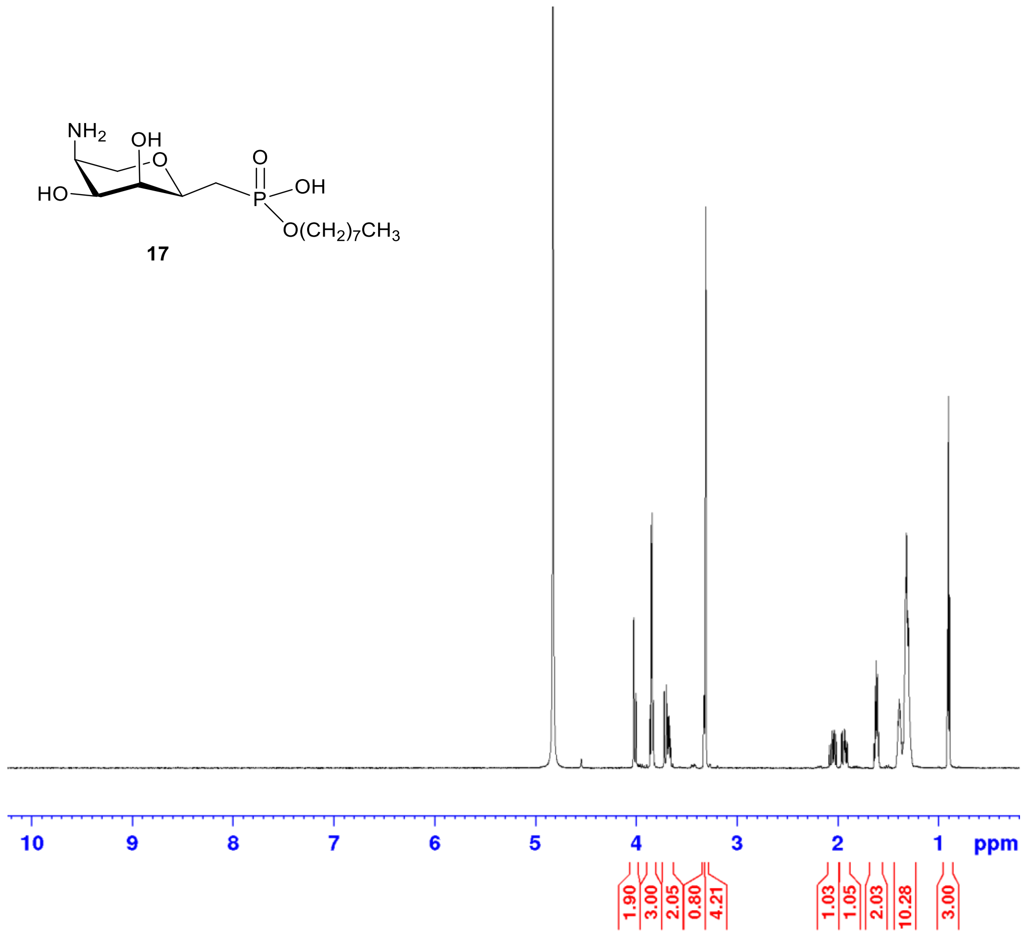
NH2
OH
O
HO
O
P
OH
O(CH2)7CH3
17
10
9
8
7
6
5
4
3
2
1
ppm
1.90
3.00
2.05
0.80
4.21
1.03
1.05
2.03
10.28
3.00

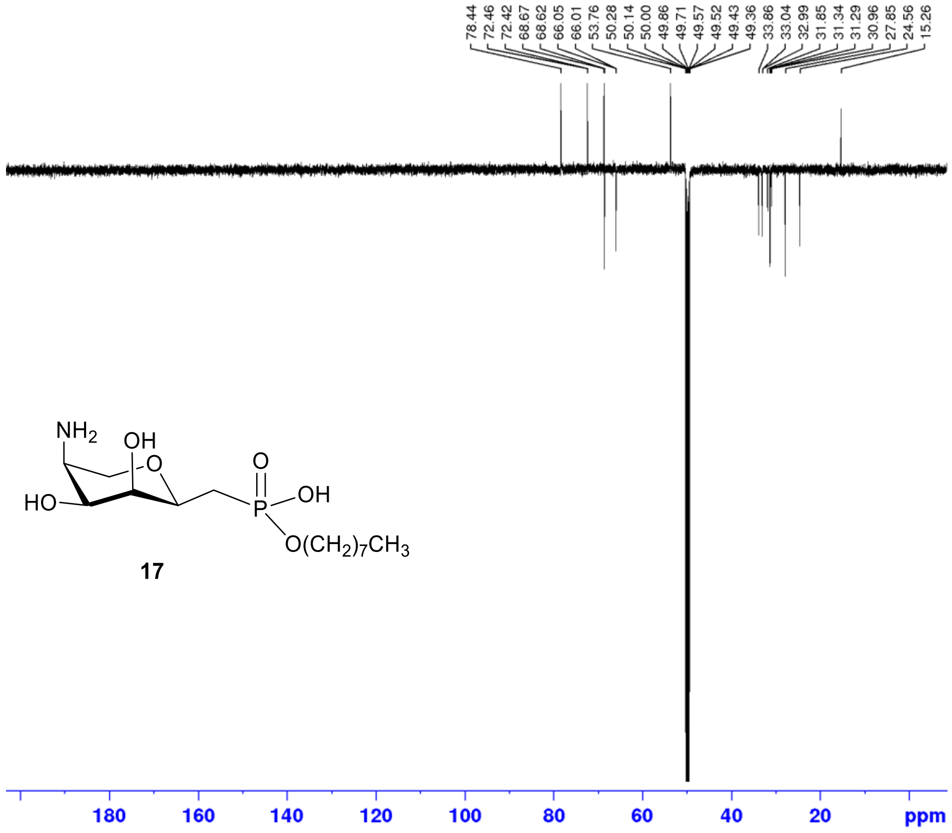
78.44
72.46
72.42
68.67
68.62
66.05
66.01
53.76
50.28
50.14
50.00
49.86
49.71
49.57
49.52
49.43
49.36
33.86
33.04
32.99
31.85
31.34
31.29
30.96
27.85
24.56
15.26
NH2
OH
O
HO
O
P
OH
O(CH2)7CH3
17
180
160
140
120
100
80
60
40
20
ppm

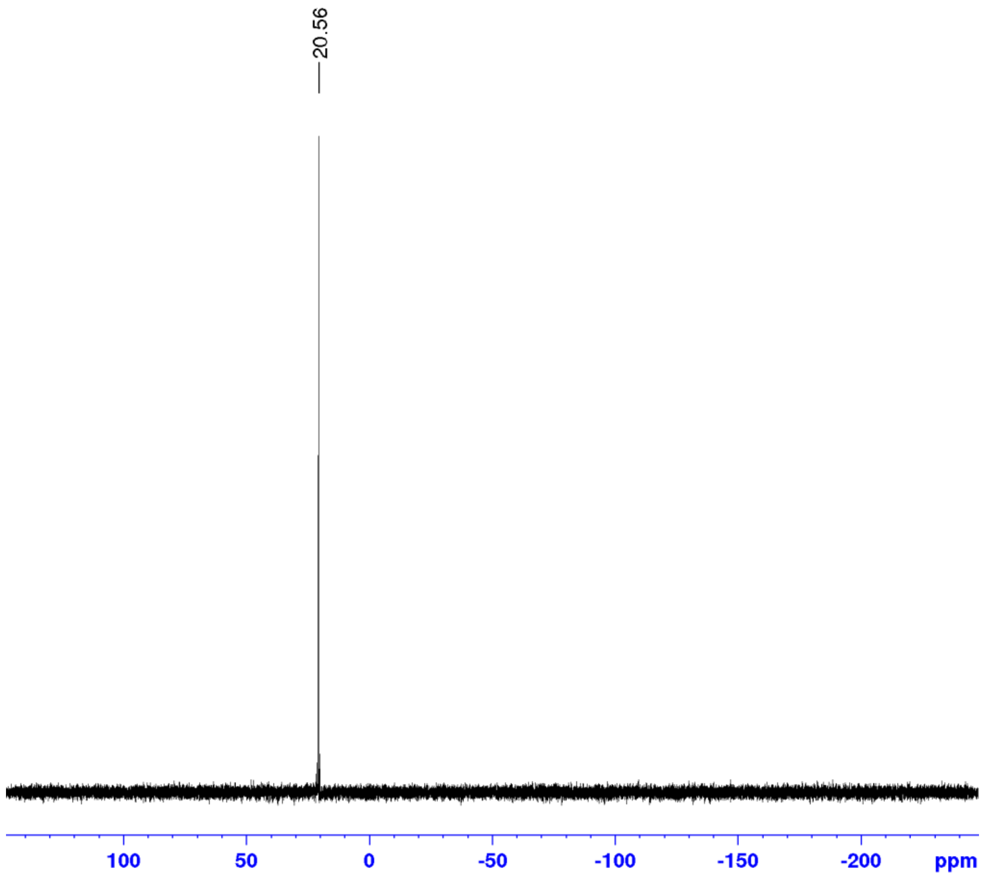
20.56
100
50
0
-50
-100
-150
-200
ppm
